# The interplay of brain neurotransmission and mental fatigue: A research protocol

**DOI:** 10.1371/journal.pone.0310271

**Published:** 2024-09-10

**Authors:** Y. Laurisa Arenales Arauz, Jelle Habay, Tjasa Ocvirk, Ana Mali, Suzanna Russell, Uros Marusic, Kevin De Pauw, Bart Roelands

**Affiliations:** 1 Faculty of Physical Education and Physiotherapy, Human Physiology and Sports Physiotherapy Research Group, Vrije Universiteit Brussel, Brussel, Belgium; 2 Research Foundation Flanders (FWO), Brussels, Belgium; 3 LIFE Department, Vital Signs and Performance Monitoring Research Unit, Royal Military Academy, Brussels, Belgium; 4 Institute for Kinesiology Research, Science and Research Centre Koper, Koper, Slovenia; 5 Faculty of Health Sciences, Sports Performance, Recovery, Injury and New Technologies Research Centre (SPRINT), Australian Catholic University, Brisbane, Queensland, Australia; 6 Australian Institute of Sport, Bruce, Australian Capital Territory, Performance Services, Canberra, Australia; 7 Sport Performance Innovation and Knowledge Excellence (SPIKE), Queensland Academy of Sport, Nathan, Queensland, Australia; 8 Department of Health Sciences, Alma Mater Europaea University, Maribor, Slovenia; 9 BruBotics, Vrije Universiteit Brussel, Brussels, Belgium; Himeji Dokkyo University, JAPAN

## Abstract

**Introduction:**

Mental fatigue (MF) significantly affects both cognitive and physical performance. However, the precise mechanisms, particularly concerning neurotransmission, require further investigation. An implication of the role of dopamine (DA) and noradrenaline (NA) is stated, but empirical evidence for this theory still needs to be provided. To address this gap, we aim to investigate the role of brain neurotransmission in elucidating if, and how prolonged cognitive activity induces MF and its subsequent impact on cognitive performance.

**Methods:**

This study (registration number: G095422N) will adopt a randomized cross-over design with sixteen healthy participants aged 18–35 years. The sessions include a familiarization, two experimental (DA: 20mg Methylphenidate; NA: 8mg Reboxetine) conditions, and one placebo (lactose tablet: 10mg) condition. A 60-minute individualized Stroop task will be used to investigate whether, and how the onset of MF changes under the influence of reuptake inhibitors. Attention and response inhibition will be assessed before and after the MF-inducing task using a Go/NoGo task. The integration of physiological (electroencephalography, heart rate), behavioral (attention, response inhibition), and subjective indicators (scales and questionnaires) will be used to detect the underlying mechanisms holistically. Data analysis will involve linear mixed models with significance at p<0.05.

**Discussion:**

The integration of diverse techniques and analyses offers a comprehensive perspective on the onset and impact of MF, introducing a novel approach. Future research plans involve extending this protocol to explore the connection between brain neurotransmission and physical fatigue. This protocol will further advance our understanding of the complex interplay between the brain and fatigue.

## 1. Introduction

Mental fatigue (MF) is a multifaceted psychobiological state characterized by a sensation of tiredness and lack of energy [1,2]. It is commonly experienced during extended periods of demanding cognitive tasks, which can lead to impaired cognitive [3–5] and/or physical performance [6–8]. There is a wide range of causes that can trigger MF, such as prolonged cognitive work, medical conditions, lifestyle or work-related issues, in addition to emotional challenges and stress [9,10]. These causes indicate that MF is a symptom that can be experienced by the general population, ranging from healthy children to adults, athletes, and clinical populations.

Various phenomena across literature, such as ego depletion, cognitive fatigue, self-control depletion, or mental strain, exhibit similarities to MF [2]. The diverse definitions and research methods associated with MF lead to inconsistent findings regarding its onset and consequences [11]. In this research, we anticipate MF to arise after prolonged and demanding cognitive tasks, evident in subjective and neurophysiological parameters. As fundamental research regarding this state is still lacking, we hope to delve further into this aspect. These insights are vital for developing effective strategies to mitigate the adverse effects of MF on physical and/or cognitive performance [1,2].

Various hypotheses and speculations attempt to explain the mechanisms of MF and its subsequent effects. MF is rooted in the intricate interplay of neural networks and neurochemical processes within the brain [1,12–18]. Several studies have utilized electroencephalography (EEG) during mentally fatiguing tasks. A recent meta-analysis conducted by Tran et al. [17] underscored a consistent increase in theta and alpha power during MF. Additionally, event-related potentials (ERPs) have been a focal point of investigation. Across studies, a decline in the amplitude of N1 [4], N2 [19], and P3 [20] components has been observed with prolonged task engagement. Such observations suggest a potential top-down modulation of sensory processing for N1, reduced cognitive control for N2, and diminished attentional resources for P3. The precise role of brain neurotransmitters, such as dopamine (DA) and noradrenalin (NA), influencing these phenomena remains to be elucidated [15].

Brain DA plays a central role in reward, motivation, cognition, and motor control [21] and is the most commonly proposed substrate in relation to MF. This theory emerges from the ideas of Pageaux et al. [22,23], who stated that prolonged mental exertion could induce an accumulation of the neuromodulator adenosine, resulting in an increased perception of effort during subsequent exercise. Martin et al. [24] further extended this hypothesis by stating that the accumulation of adenosine hinders the release of DA in the anterior cingulate cortex, thereby impairing motivation or the willingness to exert effort. This notion also aligns with the suggestion that a possible reduction of extracellular brain DA contributes to MF by negatively affecting effort/reward evaluations and motivated behavior [13,25]. However, these theoretical hypotheses require further confirmation through robust empirical evidence.

More broadly, the manipulation of neurotransmitter systems through targeted reuptake inhibitors or supplements has been used extensively to manipulate extracellular brain neurotransmitter levels in exercise science research [26]. Moeller et al. [27] demonstrated the involvement of the dopaminergic midbrain in sustaining motivation during MF by using an indirect DA agonist (methylphenidate) in healthy subjects. Furthermore, studies utilizing caffeine support its efficacy in reducing physical and cognitive performance declines associated with MF [28–31]. Caffeine has been shown to act as an adenosine antagonist that indirectly elevates brain DA levels [32]. The more profound exploration of the role of these, and other neurotransmitters, remains imperative for MF research [15]. Notably, research in the field of exercise-induced fatigue evidences the involvement of the noradrenergic brain neurotransmitter system in the onset of physical fatigue [33–36]. Increased concentrations of brain NA negatively impact physical performance capacity, accelerating the onset of physical fatigue [33–36]. These findings emphasize the complex involvement of brain neurochemistry in regulating fatigue. However, fundamental research on brain neurotransmission in MF remains limited.

Accordingly, this distinctive protocol employs a comprehensive research approach with the aim to target the proposed mechanisms of MF. we aim to investigate the role of brain neurotransmission in elucidating if, and how prolonged cognitive activity induces MF and its subsequent impact on cognitive performance. To achieve this, we will investigate subjective, behavior, and neurophysiological indicators. Based on previous findings [27–36], we anticipate a reduced onset and impact of MF following DA administration as opposed to a control condition. This may be reflected as lower perceived MF, less decline in ERPs, decreased alpha and theta band power, and improved cognitive performance with faster reaction times and higher accuracy. The opposite effect is expected for the administration of reuptake inhibitors targeting NA. The exact role of neurotransmitters on other brain dynamics [4,17,19] linked to MF remains explorative.

## 2 Materials and methods

The following protocol was made in accordance with the Standard Protocol Items: Recommendations for Interventional Trials (SPIRIT) recommendations of 2013 (S1 File) [37]. The current protocol is registered on ClinicalTrials.gov with protocol record number G095422N and identifier NCT05880342. The revised protocol (version 2), denoted as protocol number (2022-002836-30) received approval on March 15, 2023, by the Medical Ethics Committee of UZ Brussels (S2 File). Protocol alterations will be communicated to the Commission of Medical Ethics through amendments. All these amendments will be submitted through the DYCOFLOW system and subjected to approval by the commission. The proposed changes will only be implemented after the official approval.

### 2.1 Status and timeline

**Fig 1:**
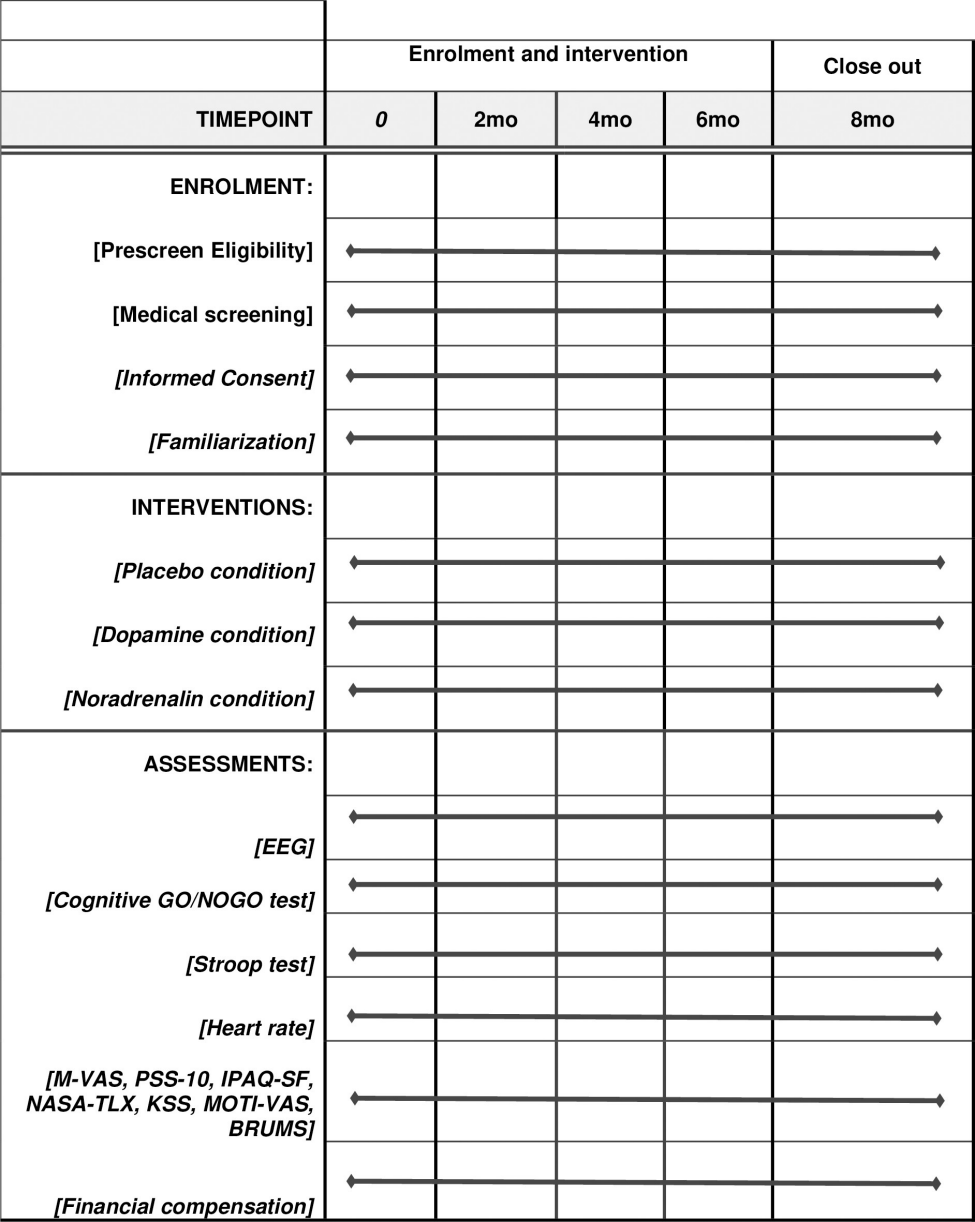
Schedule of enrolment, interventions, and assessments (SPIRIT 2013). *Notes*. Enrolment, interventions, and assessments will be assessed throughout the measuring period.

Fig 1 presents an overview of the study period. Recruitment commenced in April 2023, with the first participant familiarized with the protocol in May 2023. Data collection will conclude once we have successfully recruited 16 participants (8 male, 8 female). The objective is to complete data collection within approximately eight months (February 2024). Subsequently, analysis and results will be shared with the broader public through the publication of research manuscripts in reputable peer-reviewed scientific journals and presentations at conferences.

### 2.2 Study design and setting

**Fig 2:**
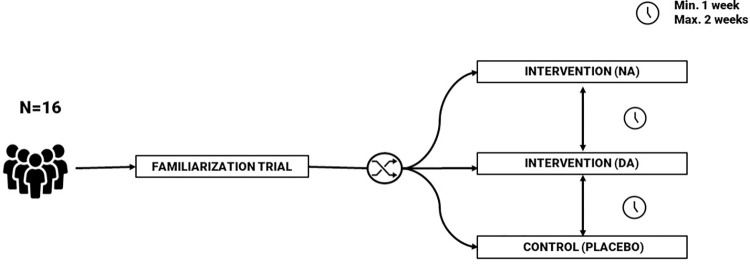
Overview of the experimental design.

The trial adopts a quasi-balanced, randomized, placebo-controlled, triple-blinded, cross-over design. Each experimental session comprises four consecutive trials encompassing one familiarization, two experimental conditions, and one control condition (Fig 2). In the experimental trials, reuptake inhibitors of DA and NA tablets will be administered, while a placebo tablet will be utilized in the control condition. The assignment of conditions (DA, NA, placebo) will be counterbalanced to mitigate the influence of order effects. To ensure adequate washout periods and minimize carryover effects, a minimum of one week and a maximum of two weeks will be targeted between each trial. The trials will be conducted within the cognitive research laboratory at research group MFYS (Vrije Universiteit Brussels, Generaal Jacqueslaan 271). Trained staff (pre- and postdoctoral level) will undertake and supervise all experimental trials.

### 2.3 Participants

#### Subjects

Sixteen healthy male and female subjects aged 18–35 years will be recruited to participate in the study. Recruitment strategies will involve word-of-mouth promotion, flyer distribution, and utilization of social media platforms. Participants will complete an online pre-screening questionnaire in the Research Electronic Data Capture (REDCap) system (see "Data collection and management”) with specific eligibility criteria outlined. The specific eligibility criteria are displayed in Table 1. Recruited participants who meet the eligibility criteria established via screening questionnaires will receive the participant information form. Information folder that thoroughly outlines the specifics of our research.

**Table 1:** Participant criteria.

Inclusion criteria	Exclusion criteria
Aged 18–35 years	Pregnancy
Healthy male and female individuals (exercise ≥1 x week)	Allergies to specific substances utilized in the current study (lactose, gluten)
No use of medication ^1^	Injuries of any kind in the past 6 months that hinder participation
No indication of risk in general fatigue, depression, and burn-out^2^	History of suffering from any mental/psychiatric disorder
Current non-smoker	Participating in any concomitant care or research trials
	Suffering from color vision deficiencies
	Suffering from any chronic health condition diagnosed by a medical professional

^1^ No chronic use of medication / no prescribed medication between or before trials / no use of non-prescribed occasional medication 24 hours prior to each trial. All forms of contraceptives are allowed.

^2^Indicated by a total score of ≥ 2.59 on the Burn out assessment tool (BAT) [38]; >57 on the Multidimensional fatigue inventory (MFI) [39,40]; >16 on the Beck depression inventory-II (BDI-II) [41].

#### Safety considerations

To ensure the safety of all participants, we will implement a comprehensive risk minimization protocol. Prior to participating in the study, all potential participants will undergo a thorough medical evaluation conducted by an experienced medical doctor. This assessment is designed to identify any pre-existing medical conditions or risk factors that might affect their eligibility for the study. If a potential medical condition or risk factor will be identified in a particular participant, they will be excluded from participation. Additionally, a qualified medical doctor will be on-site for each trial, to offer immediate assistance and guidance in case of any adverse reactions to the ingestion of NA or DA. Our safety reporting procedures have been developed in alignment with established templates from the University Hospital Brussels (UZ Brussels). These standardized adverse event forms are specifically designed to record and track any safety-related incidents or observations during the study.

#### Randomization and blinding

All participants will be required to perform all three experimental conditions (NA, DA, Placebo). The sequence of treatments (one, two, three) will be randomized and quasi-balanced using a web-based computer program (www.randomization.com) to account for potential order effects (S3 File). The pharmacy of UZ Brussels will assign medications (NA, DA, placebo) to a specific treatment number (1,2,3) within the allocation list. The complete list will be shared with two unrelated researchers from the broader research group, who are not involved in the execution of the project. This process will contribute to a triple-blinded design, effectively blinding the researchers, data analysts, and participants. Treatment allocation will only be communicated to the supervising medical specialist in cases of medication-induced adverse events or upon specific requests of participants. Unblinding procedures will be conducted after completion of data analysis.

### 2.4 Data collection and management

#### Measurement devices

To continuously measure brain activity, we will use an electroencephalography (EEG) amplifier device (LiveAmp, Brain Products Munich, Germany) equipped with 64 (2x32 channels) active electrodes attached to the LiveAmp actiCAP adapter. These electrodes will be positioned on the participants’ scalp (Acticap slim/snap, Brain Products Munich, Germany) in accordance with the “10:20 International System” [42]. The sampling rate will be configured at 500 Hz (Brain Vision Recorder, Brain Products, Munich, Germany) while ensuring that electrode impedance remains below 25 kΩ. The visual stimuli and collection of cognitive task responses will be facilitated through triggers embedded within E-Prime 3.0 software (Psychology Software Tools, Pittsburgh, PA) via a trigger box. Heart rate measurements will be obtained using the Polar H10 (Polar Electro Finland), and the administration of questionnaires will be conducted online via REDCap using an electronic tablet.

#### Outcome measures

**Fig 3:**
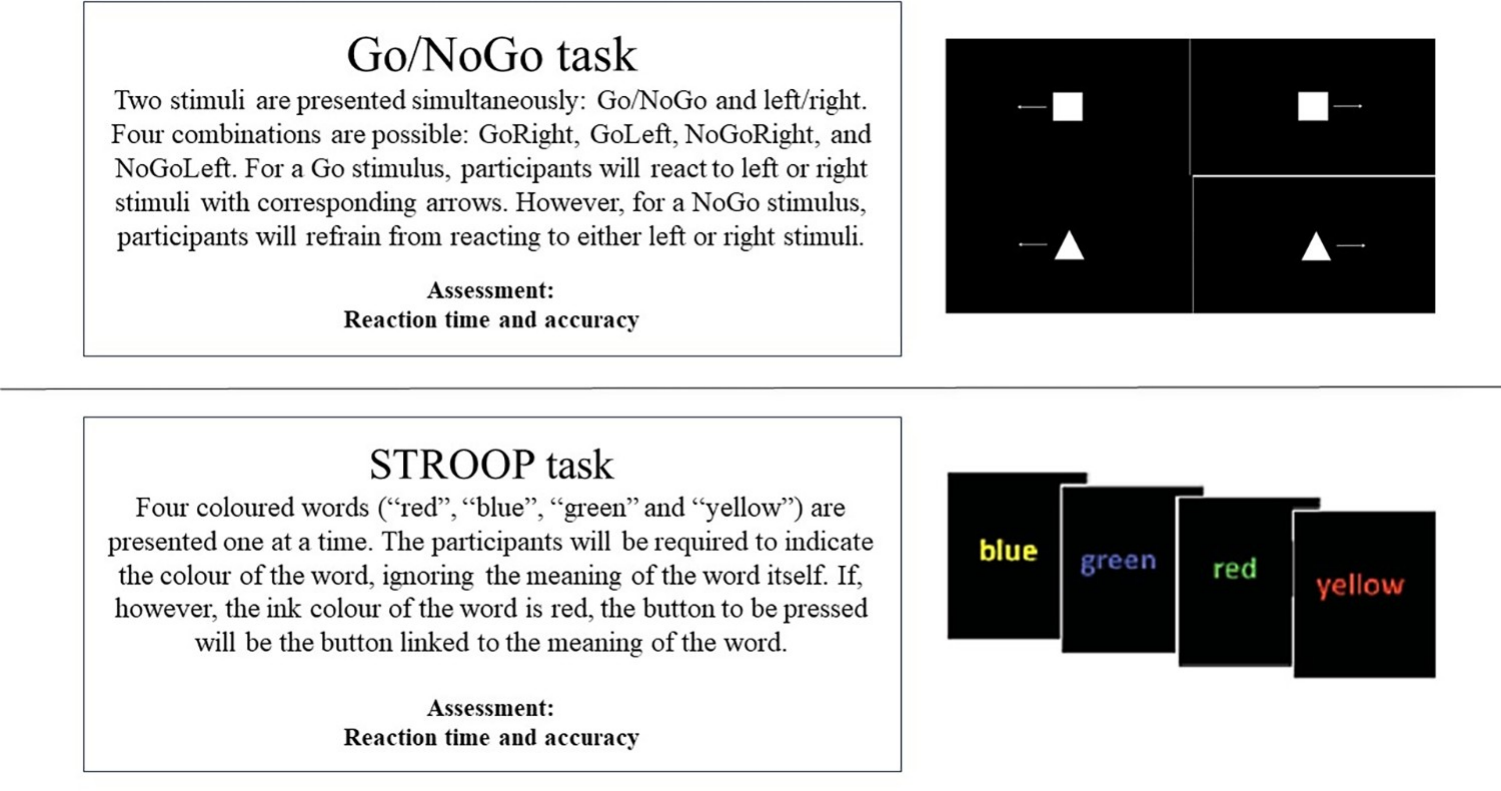
Cognitive tasks assessed in the current protocol.

This protocol will include multiple physiological, behavioral and subjective measures during cognitive tasks (Table 2, Fig 3). For primary outcome measures, we will assess brain activity (EEG spectral frequency bands and visual evoked potentials), performance (accuracy and reaction time), and subjective MF (MF-visual analogue scale) during the MF-inducing task (Stroop task). Additionally, to evaluate the impact of MF on cognition, we will measure brain activity (visual evoked potentials) and performance (reaction time and accuracy) on the Go/NoGo task. Secondary outcome measures will include heart rate and self-administered questionnaires/scales that capture internal workload (NASA-tlx), sleepiness (KSS), motivation (MOTI-VAS), stress (PSS-10), and mood (BRUMS).

**Table 2:** Measurements within the study protocol.

Measurements	Description
**Physiological**	
** • Brain activity: EEG power spectral density, EEG source localization, and VEPs during cognitive tasks [continuous]** ^**1**^	**• Measured by Brain Vision Recorder**
** •** Heart rate [continuous]	**•** Measured by a heart rate monitor before, during, and after the Stroop task (Polar H10)
**Behavioral**	
** • Accuracy of cognitive tasks [continuous]** ^**1**^	**• Both Go and No/Go trials and normal and inhibitory stimuli of Stroop task will be assessed by Eprime 3.0 software**
** • Reaction time of cognitive tasks [continuous]** ^**1**^	• Go trials and normal and inhibitory stimuli of Stroop task will be assessed* by Eprime 3.0 software
**Subjective**	
** •** Sleepiness: Karolinska sleepiness scale [43] [ordinal]	**•** This scale involves a 10-points sleepiness scale from “extremely alert” to “extremely sleepy”*
** • MF: VAS scale 0–100 [44] [continuous]** ^**1**^	• This scale involves a rating of MF on a 10 cm line with “not at all” to “extremely”*
** •** Motivation: VAS scale 0–100 [44] [continuous]	**•** This scale involves a rating of motivation on a 10 cm line with “not at all” to “extremely motivated”*
** •** Mood: BRUMS [45] [ordinal]	**•** This scale features 24 mood descriptors with a 5-point Likert scale with “not at all” to “extremely”*
** •** Subjective workload: NASA-tlx rating scale [46] [ordinal]	**•** This scale is composed of six subscales assessing subjective workload*
** •** Stress: PSS-10 [47] [ordinal]	**•** The PSS-10 is a 10-item questionnaire evaluating the degree of perceived stress the last month*

^**1**^ Primary outcome measures.

Abbreviations: EEG = Electroencephalography; VEPs = Visual evoked potentials; KSS = Karolinska sleepiness scale; VAS: Visual analogue scale; MF = mental fatigue; BRUMS = Brunel Mood Scale; NASA-TLX = National Aeronautics and Space Administration Task Load Index = PSS = Perceived stress scale.

* Measured by Redcap on an electronic tablet.

#### Data storage and management

Data of secondary outcome measures will be collected and managed using REDCap (Research Electronic Data Capture, Nashvillle) hosted at [UZ Brussel] [48,49]. REDCap is a secure web-based application that includes compliance with general data protection regulations. Each participant will be assigned an exclusive identity code, which will ensure pseudonymization. Behavioral and physiological data will be securely stored on the VUB (Vrije Universiteit Brussels) SharePoint platform. Additionally, an external backup solution will be facilitated by an external hard drive. Data will be obtained for 20 years adhering to the Brain Imaging Data Structure (bids.neuroimaging.io/) within the sealed Synology database. The data stored will be kept in its original state and will be linked to the researcher’s contributor ID (ORCID) as well as the Digital Object Identifier (DOI) of the corresponding article. As for data sharing, Anonymized full datasets will be available through a public repository facilitated by our institution (VUB). This will result in FAIR (Findable, Accessible, Interoperable and Reusable) data of our study. Data in the present study will be managed confidentially, conform the general data protection regulation (GDPR) of 27 April 2016. Participating in this study will entail an agreement with the investigators to collect data and publish the subsequent results in scientific journals. Participants will maintain the right to inquire about the specific data gathered and request corrections if any errors will be identified. The participants will also retain the right to stop his/her participation in the study at any moment without reasoning.

### 2.5 Procedures

#### First visit (familiarization)

1. The participant receives a thorough explanation outlining the upcoming procedures, used terminology, and overarching objectives of the present study.
2. A medical specialist examines the participant through a system anamnesis, sportive anamnesis, familial anamnesis, medication and drug usage, general clinical examination, skinfold measurements and rest electrocardiogram.
3. The participant follows the informed consent procedure provided by YLAA.
4. The participant familiarizes with all questionnaires and cognitive tests in a sequence mirroring the actual experimental trials. The familiarization of the Stroop task consists of a determination of the participants’ Stroop level to customize the intervention task [50,51]. This task is divided into 96-stimulus bocks, with accuracy calculated after each block. When accuracy exceeds 85%, difficulty will be adjusted by reducing the stimulus presentation time (SPT). The SPT decreases in the following order: 1100, 1000, 900, 800, 700, 600ms. If the SPT falls below 85% accuracy, the respective trial is designated as erroneous, prompting the subject to repeat the trial while maintaining the SPT. The trial is terminated if the participant makes three consecutive errors or accumulates five errors in total. The SPT of the last successfully completed block is considered as the maximum capacity of that individual and will be used to individualize the difficulty of the 60-minute Stroop task in the following trials.
5. The participant is debriefed and scheduled for subsequent experimental appointments.
6. The researcher distributes a pre-test checklist via email 48 hours in advance of each trial. The purpose of this checklist is to remind participants to remain adherent to the eligibility criteria throughout the trials (no use of medication, no smoking etc). In addition, participants will be asked to eat a similar dinner, avoid the use of caffeine, and limit heavy exercise or activity efforts 24 hours prior to the trial.

#### Experimental trials

**Fig 4:**
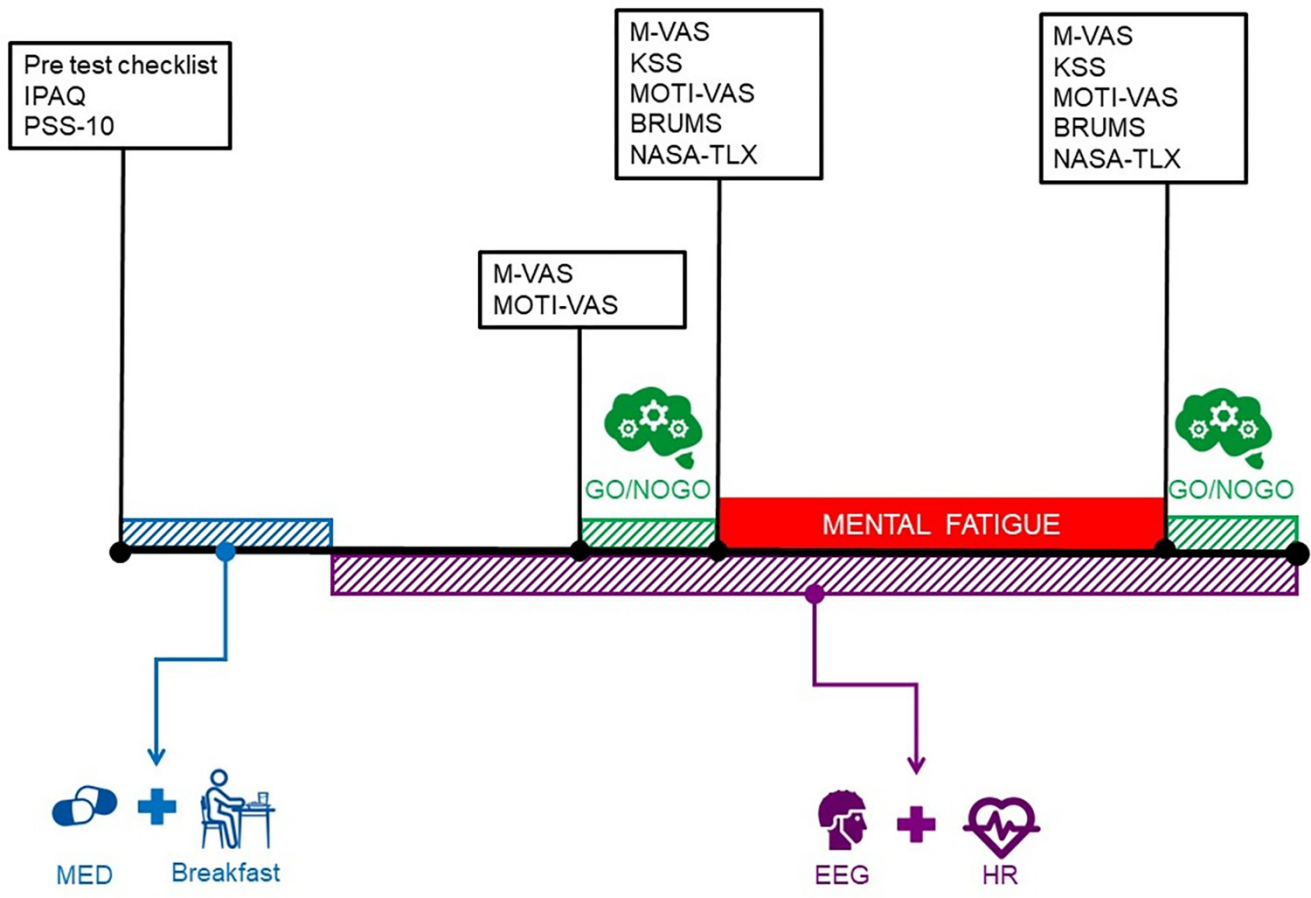
Overview of the procedures within the experimental trials. Abbreviations: MED = Medication; IPAQ-SF = international physical activity questionnaire short form; PSS = Perceived stress scale; M-VAS = Mental fatigue VAS-scale; KSS = Karolinska sleepiness scale; MOTI-VAS = Motivation VAS-scale; BRUMS = Brunel Mood Scale; NASA-TLX = National Aeronautics and Space Administration Task Load Index; EEG = Electroencephalography; HR = Heart rate.

Fig 4 provides an overview of the entire experimental/control trials.

1. The researcher administers the pre-test checklist verbally
2. The participant ingests the medication (see Table 3 for medication details).
3. The participant completes the perceived stress scale (PSS-10) and the short version of the International Physical Activity Questionnaire-Short form (IPAQ-SF) [52] to check the level of activity in the past seven days.
4. The participant digests the medication while they do a relaxing activity (e.g. watching a show, reading a book) by choice.
5. After one hour, the participant consumes a standardized breakfast (S4 File).
6. The researcher equips the participant with an the EEG cap and a heart rate monitor. Brain activity will be measured throughout the trial, whereas heart rate will be noted before, during (only for each block of the Stroop task), and after each cognitive task.
7. The researcher records a baseline EEG measurement for four minutes, with two minutes each for eyes open and eyes closed. This baseline captures the participant’s resting-state brain activity and serves as a reference for detecting any changes during subsequent tasks. During the baseline, participants should minimize head movement, blinking, frowning, and any contact with their head to avoid noise/artifacts.
8. The participant completes the MF state and motivation scales.
9. The participant completes the pre-cognitive task (Go/NoGo).
10. The participant completes the pre-fatigue states of perceived MF, motivation, mood, MF, workload, and sleep.
11. The participant performs the MF-inducing intervention (± 60 min Stroop task at a predetermined level).
12. The participant completes the post-fatigue states of perceived MF, motivation, mood, MF, workload, and sleep.
13. The participant completes the post-cognitive task (Go/NoGo).
14. The researcher takes a second baseline EEG procedure for four minutes with eyes open (two minutes) and eyes closed (two minutes).

**Table 3:** Overview of medications and dosages utilized in the present study.

Medication	Pharmacological substance	Type	Main targets	Total dosage
Ritalin 10 mg	Methylphenidate	Neurotransmitter reuptake inhibitor	DA	2 pills (20 mg)
Edronax 4 mg	Reboxetine	Neurotransmitter reuptake inhibitor	NA	2 pills (8 mg)
P-Tabletten weiß 7 mm Lichtenstein 5mg	Lactose-Monohydrate	Placebo	Lactose	2 pills (10 mg)

Abbreviations: DA = Dopamine; NA = Noradrenaline.

^a^ Both a DA and NA reuptake inhibitor.

### 2.6 Statistical methods

#### Sample size calculation

A priori power analysis was conducted based on the results reported by Van Cutsem et al. 2022 (F (1,18) = 8.2; p = .010; ηp2 = .313) [53]. The reference study utilizes a comparable experimental design, participant population, and cognitive targets. Additionally, the underlying cognitive processes (response inhibition and cognitive control) targeted by the tasks are conceptually aligned. The sample size for this study was determined using G*Power 3.1 Software [54]. The input parameters included a statistical test of MANOVA repeated measures within factors, a significance level of 0.05, a power of 0.95, and 3 measurements. Based on these parameters, the analysis indicated a sample of at least 15 subjects. We will include 16 participants to ensure a gender-balanced sample with equal proportions of male and female participants. This number also accounts for potential data loss due to unusable EEG recording caused by noise or technical issues.

#### EEG data (pre)processing

For EEG data analysis, an automated preprocessing pipeline [55,56] specified for each analysis (spectral analysis, ERPs, source localization) will precede machine learning processes. EEGLAB will be used to process the data further. All EEG data will be preprocessed and analyzed using the steps in the EEGLAB toolbox outlined in Delorme and Makeig (2004) [57] within the MATLAB software (The MathWorks, Inc., Natick, MA, United States, version MATLAB R2024a). We will use the standard 64-channel location, including the reference and the ground electrode (actiCAP, Brain Products gmbH, Gilching, Germany).

We will conduct EEG spectral analysis to discern variations in energy across various frequency components. Changes in spectral power will indicate alterations in brain activity related to cognitive tasks [58]. We plan to use the Fast Fourier Transform model to identify the power or amplitude of different frequency bands associated with various physiological and cognitive states within the EEG signal. Although we will consider all frequency bands, Tran et al. [17] identified the most consistent and significant alterations in alpha and theta wave activity, warranting a focused analysis of these frequency bands in our study.

Event-related potentials are time-locked modulations in the brain’s electrical activity corresponding to specific sensory, motor, or cognitive event [5]. They arise as evoked responses within EEG recordings, reflecting the brain’s reaction to specific stimuli. With prolonged task engagement, a decrease has been observed in the amplitude of visual evoked potentials in N1 [4], N2 [19], and P3 [20] components. While all components of visual evoked potentials during cognitive tasks will be examined, our primary focus will be on the N1, N2, and P3 components.

Source localization will be employed to estimate the brain regions where activity originates, based on EEG signals. Our aim is to investigate how the spatial distribution of brain activity shifts during various cognitive tasks or in response to stimuli. The proposed method for source localization is sLORETA [58,59].

#### Analysis of experimental data

The normality of the data distribution will be assessed using the Shapiro-Wilk test, augmented by visual inspection through histogram plots. We intend to employ linear mixed models (LMMs) to analyze the influence of DA and NA on the impact and consequence of MF. Given the variability in our data and the diversity of our parameters, the specific composition of the LMM will depend on the characteristics of the dataset obtained. Nonetheless, our analysis will focus on primary outcome measures that are continuous in nature. We further anticipate to include both fixed and random factors in our LMM analysis. Fixed factors will encompass "Condition” (NA/DA/placebo) and “Time”. Random factors will be used to account for individual variability and other sources of random variation within the dataset.

For secondary outcome measures, each scale (BRUMS, NASA-TLX, MOTI-VAS, PSS-10, KSS, Table 2) will be appropriately prepared and assessed by calculating mean scores for subscales (e.g., NASA-TLX, BRUMS) or sum scores (e.g., PSS-10, KSS) for each time instance. Next, a repeated measures ANOVA or a non-parametric alternative (Friedman test for data of ordinal level) will be conducted using "Condition" (NA, DA, placebo) and "Time" as within-subject factors to determine whether significant differences exist between conditions and across time points.

Data exploration, visualization, and statistical tests will be performed using R (version 4.1.2) [60], with significance set at p<0.05. Necessary statistical software packages available in R, such as lme4 [61] for LMM, will be employed for each analyses.

## 3. Discussion

This experimental cross-over trial aims to investigate the role of brain neurotransmission in elucidating if, and how prolonged cognitive activity induces MF and its subsequent impact on cognitive performance. Outcome measures include brain activation patterns, behavioral, and subjective indicators of MF. Building upon prior research findings [27–36], our hypothesis posits a positive impact of DA on the onset and impact of MF. This means improved cognitive performance indicating lower reaction time and higher accuracy. In contrast, we expect an adverse effect of NA on cognitive performance compared to the control condition. A more thorough exploration into the precise influence of neurotransmission on MF-related brain activity patterns will provide a more detailed elucidation of the effects.

The present protocol possesses notable strengths. This will be the first study to investigate both DA and NA reuptake inhibitors in the onset and impact of MF. Prior MF research has primarily employed the use of reuptake inhibitors to explore cognitive or physical performance [26,62]. Moreover, we will implement a rigorous design to enhance the overall robustness of the research. These measures involve the implementation of triple blinding procedures, the incorporation of subjective, physiological, and behavioral measures, and the consideration of various influencing factors. This will hold significance in the field of MF research, considering the complex nature of MF influenced by a wide array of factors and interindividual differences [63].

While this protocol provides valuable insights, it is important to acknowledge some limitations. First, various methods are employed in literature to induce MF [62]. These methodologies operate under the assumption that prolonged, demanding cognitive activities are responsible for inducing this form of fatigue. However, susceptibility to MF is contingent upon numerous factors that are challenging to control [63]. The consistency of the 60-minute Stroop task in inducing MF in individuals, along with its representation of real-life situations, warrants careful consideration. However, Based on current knowledge and integrated parameters, we will assess whether, and to what extent, MF is initially induced. Secondly, boredom is not separately investigated within our protocol. It is vital to consider the potential influence of boredom on the perception of MF, as highlighted by previous research [8,64]. The sensation of boredom is among the mood states assessed in the BRUMS administered both before and after the mentally fatiguing task. Hence, this factor will be carefully considered in our study.

This research further aims to contribute to the current scientific knowledge on MF in diverse ways. One of the contributions is the confirmation or debunking of the DA theory, as mentioned by Martin et al. [24]. Furthermore, to effectively detect, prevent, and counteract the detrimental effects of MF, it is crucial to identify the central mechanisms of MF. As proposed by Habay et al. [1], directing fundamental research efforts toward understanding brain mechanisms can significantly benefit both applied (sport-specific) and clinical science. Targeted interventions may be developed to boost cognitive resilience in those prone to MF, such as athletes, high-stakes professionals, and patients with cognitive impairments. The current protocol further underscores the significance of transparent reporting methods within MF studies contributing to transparency within the broader research community. Lastly, the present protocol serves as a foundation for future research. The involved research group intends to implement a similar protocol to investigate the link between brain neurotransmission in physical fatigue and the subsequent effects of MF on physical fatigue.

## Supporting information

S1 File Addressed items of the current protocol according to the SPIRIT 2013 checklist. (PDF)

S2 File Approved experimental study protocol. (PDF)

S3 File Randomization of medication order. (PDF)

S4 File Standardized breakfast for each participant. (PDF)
