# Supplementary material for: The interplay of brain neurotransmission and mental fatigue: A research protocol
Source: PLoS One. 2024 Sep 10;19(9):e0310271. doi: 10.1371/journal.pone.0310271 (PMC11386444; doi:10.1371/journal.pone.0310271)
Supplement: S2 File — (PDF) [file pone.0310271.s002.pdf]

EXPERIMENTAL TRIAL STUDY PROTOCOL

Title of the project

THE ROLE OF THE BRAIN IN MENTAL AND PHYSICAL FATIGUE

Investigator(s)	Date
Prof. Dr. B. Roelands (VUB (MFYS))	30/11/2022
(Head investigator and Head of Research Group)	
Prof. Dr. K. De Pauw (VUB (MFYS))	30/11/2022
Prof. Dr. Uros Marusic (ZRS)	02/12/2022
Drs. J. Habay (VUB (MFYS))	30/11/2022
Drs. Yahaira Laurisa Arenales Arauz (VUB (MFYS))	02/12/2022
Drs. Tjaša Ocvirk (VUB (MFYS))	02/12/2022

Departments/laboratories involved in the study

Human Physiology and Sports Physiotherapy Research Group (MFYS), Vrije Universiteit Brussel, Brussels, Belgium

Protocol log:

Version 2, date: 05/01/2023

Table of contents

1. INTRODUCTION 2

2. OBJECTIVE AND HYPOTHESES 5

3. MATERIALS & METHODS 5

3.1. RESEARCH DESIGN, TIMELINE AND SETTING 5
3.2. SUBJECT ELIGIBILITY CRITERIA AND RECRUITMENT 7
2.2.1 INCLUSION 7
2.2.2 EXCLUSION 7
2.2.3 RECRUITMENT 7
3.3 EXPERIMENTAL PROCEDURE AND INTERVENTION/CONTROL TASK 8
3.3.2. PHARMACOLOGICAL SUBSTANCES: 13
3.3.3. PLACEBO: 13
3.4 PRIMARY OUTCOME MEASURES 13
3.4.1 PERFORMANCE MEASURES 13
3.4.2 EEG AND EMG 14
3.5. SECONDARY OUTCOME MEASURES 15
3.5.1. QUESTIONNAIRES DURING THE TRIAL 15
3.5.2. PHYSIOLOGICAL MEASUREMENTS DURING THE TRIAL 17

4. SAMPLE SIZE, RECRUITMENT AND RETENTION INTERVENTIONS 17

5. ASSIGNMENT OF TREATMENT ORDER AND ALLOCATION CONCEALMENT 18

6. STATISTICAL ANALYSIS 19

7. DATA STORAGE AND MANAGEMENT 19

7.1. DATA STORAGE: 19
7.2. DATA MANAGEMENT: 19

8. ETHICS AND DISSEMINATION 20

REFERENCES 21

1. Introduction

Fatigue has been described as tiredness, exhaustion, lethargy or weariness. It refers to a physical and/or mental state of being tired and lack of energy. There is a wide range of causes that can trigger fatigue such as medical conditions, lifestyle or work-space related issues, but also emotional concerns and stress. These causes indicate that fatigue (be it mental or physical) is a symptom that can be experienced in the general population, ranging from healthy children over hard-working adults, athletes, and patient populations. Although it is such a well-known condition, still a lot of questions remain about its mechanism and potential interactions between mental and physical fatigue. The current project progresses on the current knowledge to further unravel the complex central triggers of fatigue during mental or physical effort.

Exercise-induced physical fatigue (PF) is a multidimensional concept and a distinction has been made between central and peripheral fatigue. Peripheral fatigue involves impairments located in the muscle, while central fatigue is defined as a failure of the nervous system to drive the muscle maximally (1). There is fairly strong evidence that the central component of exercise-induced fatigue, observed through changes in performance and/or increased ratings of perceived exertion (RPE) is caused by changes in the synthesis and metabolism of brain monoamines, such as serotonin (5-HT), dopamine (DA) and noradrenaline (NA) (2-4). However, it remains to be determined which brain regions are primarily involved in the onset of fatigue.

Mental fatigue (MF) can be defined as *"a psychobiological state that arises during prolonged demanding cognitive activity of any kind, and results in an acute feeling of tiredness, a decreased performance capacity and/or a difference in brain activation"(5-8)*. Because of the high prevalence of MF, a multitude of studies have assessed how MF can impact different kinds of human performance (9). These studies have shown that MF negatively affects important aspects of both cognitive (e.g. attention (10) and planning (11)) and physical performance (e.g. endurance (12, 13) and sport-specific psychomotor performance (5)). Because of its effects, some researchers have formulated theoretical mechanisms of the effect of MF on physical performance (14). Interestingly, the reduction in physical performance in a mentally fatigued state is not mediated by an exacerbation of peripheral factors normally associated to PF (7). The negative effect of mental fatigue on exercise performance is attributed to a higher-than-normal RPE (12). Furthermore, it has been argued that MF may act on brain neurotransmission, with an increase in DA concentration as the most potent candidate to decrease MF by possibly sustaining effort during task performance (15-18). Decreased DA levels, as expected in mentally fatigued subjects, might result in elevated noise levels, negatively affecting information processing (19). Martin et al (20) also argued that MF could result in an increase in cerebral accumulation of adenosine (an inhibitory neuromodulator of dopamine) leading to a higher perception of effort and decrease in dopamine release. This argument is further strengthened by a study showing that a caffeine-maltodextrine mouth rinse (adenosine receptor antagonist) reduced the development of MF (21). Further studies are however required to confirm which brain neurotransmitters are potentially

involved in the negative effects of MF on subsequent exercise capacity and cognition. To more profoundly study the brain alterations induced by MF during a subsequent physical exercise task, electroencephalography (EEG) is a promising technique (22). Some studies have already used EEG during a mentally fatiguing task. A recent meta-analysis paper by Tran et al. (8) highlighted that an increase in theta and alpha power has been consistently observed in the presence of MF. Also event-related potentials (ERPs) have been studied. Mental fatigue decreased the amplitude over time for the N100, N200 and P300. This could be interpreted as reflecting a top-down modulation of sensory processing for the N100 (23), a decrease in cognitive control for the N200 (24), and a decrease in attention for the P300 (25). Investigating the role of neurotransmitters in these phenomena is yet to be determined. Finally, motor related cortical potential (MRCP) amplitude is directly linked to the intensity of muscle contraction and has been extensively used in the literature as an index of the central motor command, closely associated to the generation of the RPE (26). It is hypothesized that an increased amplitude of the MRCP reflects the increased motor demand required to perform a physical task in a mentally fatigued state, but much more research is required to confirm this hypothesis.

A major gap in knowledge exists regarding the interaction between physical and MF. Due in part to methodological challenges in measuring brain activation during exercise, we do not know how brain activity changes during exercise in a mentally fatigued state, and in which cortical areas these changes arise. Brownsberger et al. (2013) (27) demonstrated that individuals with higher self-reported sensations of fatigue (induced by a mentally fatiguing task) produced elevations of EEG beta activity in the prefrontal cortex prior to exercise. Very recently, Jacquet et al. (2021) (22) took a first step in answering this question by investigating changes in MRCP during actual and imagined isometric contractions of the right knee extensor muscles in a mentally fatigued state. The authors reported a clear increase in MRCP during imagined contractions, suggesting that more effort was required to perform the imagined contractions, but were unable to distinguish significant differences in MRCP amplitude, which, for them, is caused by the progressive built-up of peripheral fatigue during exercise. They concluded that future studies should tailor the physical exercise to minimize the exercise-induced decrease in force production capacity (22). In order to solve the above-mentioned data analysis issues, we aim to bring together two common electrophysiological signals, EEG and electromyography (EMG), to investigate the characteristics of fatigue onset. Since neurophysiological signals (both EEG and EMG) reflect thousands of simulations of ongoing activities, the acquired data represent a complex signal that requires appropriate feature extraction methods for the studied task. For this reason, we propose the integration of behavioral variables directly into the analyses of EEG-reconstructed brain dynamics to allow direct linkage. To this end, we plan to apply machine learning methods capable of statistically "learning" linear and non-linear relationships between brain dynamics and behavior (28-30). A central goal of these analyses is to identify a number of functionally different large-brain networks that represent different aspects of executed and recorded behavioral information. This makes it possible not only to statically

map brain activity in relation to the given task, but also to characterize specific large-scale network dynamics and describe their time-resolved properties in relation to behavior (31).

2. Objective and hypotheses

The current project aims to improve our understanding of the fundamental mechanism of physical and MF, and how MF affects PF. We aim to determine the role of brain neurotransmission in the onset of fatigue, identify the brain areas involved, and determine how brain activity and neuromuscular efficiency changes during the onset of fatigue.

The objectives of the project are as follows:

- To experimentally assess the role of a DA, and a NA reuptake inhibitor in the onset of exercise-induced fatigue
- To identify changes in brain activation associated with altered PF and fatigue perception
- To experimentally assess the effect of a NA or DA reuptake inhibitor on MF, brain activation and the sources of changes in brain activation
- To experimentally assess the role of brain neurotransmitters (DA, NA) in the interaction between mental and PF from a neurophysiological perspective.

3. Materials & methods

The following protocol was made in accordance with the Standard Protocol Items: Recommendations for Interventional Trials (SPIRIT) recommendations of 2013 (32). We intend to register the present trial at the Clinicaltrials.gov registry.

3.1. Research design, timeline and setting

The present protocol details the study design of three different experimental studies that are performed within the context of an approved FWO Weave project (G095422N). These studies all feature the same experimental design, namely a randomized, single-blinded, placebo controlled, counter-balanced, cross-over study design (Figure 1). Each experiment will consist of four consecutive trial moments performed in a research lab (cognitive room) at MFYS (VUB campus Etterbeek, Boulevard General Jaques 271, 1050 Elsene (Brussels). Trained staff (pre-doctoral researchers and trained master students) will be present during the experimental trials.

The familiarization, experimental and control trials will be separated by at least one week to ensure full recovery and washout. The experimental/control trials will be conducted at around the same time of day.

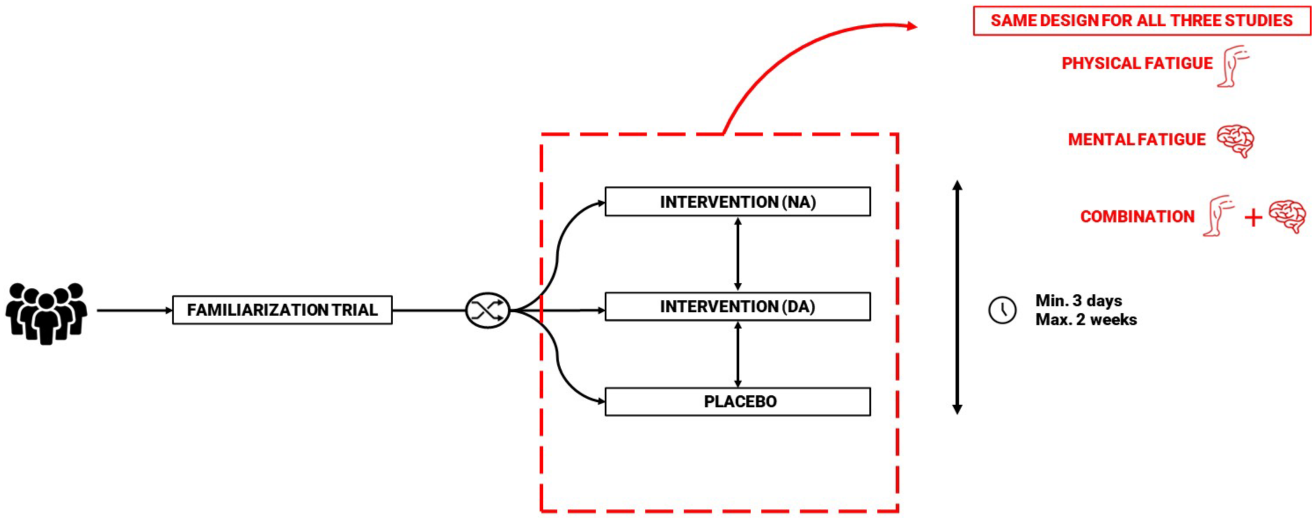

Figure 1: overview of the experimental design of the proposed protocol

3.2. Subject eligibility criteria and recruitment

3.2.1 Inclusion

- Healthy trained individuals (exercises at least 1x per week, no neurological, cardiovascular internal or musculoskeletal disorders of any kind)
- Male or female
- 18 to 35 years old
- No use of chronic or occasional prescribed medication (except for contraceptives)
- Non-smoker

3.2.2 Exclusion

- Injuries of any kind in the past 6 months
- Pregnancy
- Specific food or drink allergies (e.g. lactose and/or gluten intolerance)
- Suffering from a chronic health condition (could be neurological, cardiovascular, internal and musculoskeletal)
- Participating in any concomitant care or research trials
- History of suffering from any mental/psychiatric disorders (33)
    - Suffering from a higher risk of burn out, indicated by a total score of ≥ 2.59 on the Burn out assessment tool (BAT) (34)
    - Suffering from high general fatigue, indicated by a score of >57 on the Multidimensional fatigue inventory (MFI) (35)
    - Suffering from depression, indicated by a score of >16 on the Beck depression inventory-II (BDI-II) (36)
- Use of medication
    - Chronic use with an exception for contraceptives
    - Prescribed medication in between or before trials
    - Use of non-prescribed occasional medication 24 hours prior each trial
- Use of caffeine and heavy efforts 24 hours prior each trial
- Suffering from colour vision deficiencies
- Not eating a standardized meal, the morning of each trial

2.2.3 Recruitment

Students will be recruited through word of mouth advertising, flyers, the use of social media, contacting the subject pool with the research groups MFYS and VIPER, etc. Participants will be asked to fill in an online pre-screening questionnaire (in/exclusion criteria, BAT, MFI, BDI-II) for eligibility purposes.

When considered available and eligible, participants will be contacted to schedule their first session (familiarization trial).

3.3 Experimental procedure and intervention/control task

When arriving for the first trial (the intake/familiarization-trial), participants will get an explanation of what is expected and be told that the purpose of the research is to investigate the cerebral response during different activities with the administration of different drugs to elicit different neurotransmitter responses. Participants will have the opportunity to ask questions, and should have read and signed the informed consent prior to the start of the trial. After signing, participants will visit the medical doctor for a medical examination that exists of a system anamnesis, sportive anamnesis, familial anamnesis, medication and drug usage, general clinical examination, skinfold measurements and rest-ECG. Afterwards, the participants will also be able to familiarize themselves with all questionnaires and tests used during the study protocol. These questionnaires and tests include: the MF or PF visual analogue scale (M-VAS/P-VAS), Boredom-VAS (B-VAS), motivation-VAS (Moti-VAS), National Aeronautics and Space Administration Task Load Index (NASA-TLX 4), Brunel Mood Scale (BRUMS), Karolinska Sleepiness Scale (KSS), rate of perceived exertion 100 scale (RPE-100), the psychomotor vigilance task (PVT) or cognitive performance task (Go-NoGo task). Participants will also be familiarized with the intervention and control trial dependent on the performed study (PF: leg extension; MF: Stroop task). To avoid the induction of cognitive fatigue during the familiarization trial, breaks will be administered and different topics will be assessed interchangeably to elicit task switching (which can halt the induction of MF) (37).

The familiarization of the Stroop task consists of a determination of the participants' Stroop level, which will be determined to individualize the eventual intervention Stroop task. In this task, four coloured words ("rood", "blauw", "groen" and "geel") will be presented one at a time on a computer screen. The participants will be required to indicate the colour of the word, ignoring the meaning of the word itself. If, however, the ink colour of the word is red, the button to be pressed will be the button linked to the real meaning of the word. The word presented and its ink colour will be randomly selected by a computer (100% incongruent), with all incongruent word-colour combinations being equally common. The particular Stroop task of the familiarization trial (the Stroop max task) will be divided in blocks of 96 stimuli. After each block, the accuracy (ACC) will be calculated. When the ACC is higher than 85%, the difficulty of the Stroop task will be increased by decreasing the stimulus presentation time (SPT). If the ACC is lower than 85%, this block will be considered as an 'error' and the subject must redo the block without changing the SPT. The first block will have a SPT of 1500 ms and will have the main objective to get familiar with the task. When the required ACC is achieved, the SPT will decrease in the following order: 1100, 900, 800, 700, 650, 600, 550 ms… To determine the individualized cognitive load. Before the start of each block, the subjects will be able to take a break if they like. When the subject

makes three errors in a row, or five errors during the whole trial, the trial will end. The SPT of the last, successfully completed block will be considered as the maximum capacity of that individual and will be used to individualize the difficulty of the 60-min Stroop task. After this task, participants will be shortly debriefed and subsequent appointments will be made for the experimental and control procedures.

After arrival at the laboratory for the experimental/control trial, participants will be asked to go to the toilet. Afterwards, participants will be asked to fill in a pre-test checklist (checking adherence to pre-test protocol), a perceived stress scale (PSS-10) and the short version of the International Physical Activity Questionnaire (IPAQ) . The participant will then be given the drug (see 3.3.2. Pharmacological Substances), before or during a standardized breakfast. Participants will then be equipped with the EEG (Acticap, Brain Products Munich, Germany) and the Equivital (eqlifemonitor, Equivital, United Kingdom) or Polar H10 sensor to measure heart rate/respiratory rate.

After correct application of the EEG setup, an EEG baseline measure with 10 blinks, eyes open (2 min) and eyes closed (2 min) will be taken. During baseline EEG, participants are instructed to minimize movement of the head and eye blinking, to avoid frowning, to maintain the same posture and not to touch their head with their hands in order to minimize movement, sound and muscle artifacts. To aid the participant, the test administer will advise him/her to focus on a specific point in the distance (eyes open). Then the first set of questionnaires (i.e. M-VAS/P-VAS or both (depending on the performed trial), Motivation) will be administered. After this, the participant will perform the pre cognitive performance tasks (i.e. the Go/NoGo task or PVT). This is followed by another bundle of questionnaires (i.e. Brums, M-VAS/P-VAS, B-VAS, Motivation and the NASA-TLX), followed by the intervention/placebo/control condition of the respective experiment. Afterwards, different questionnaires will again be administered (i.e. Brums, M-VAS/P-VAS, B-VAS, Motivation and the NASA-TLX), and the post cognitive tasks will be conducted. Afterwards, another baseline of EEG will be taken replicating the first baseline procedure (10 blinks, eyes open, eyes closed). At the end the participant will have a chance to shower. A researcher will sit behind the subject to ensure compliance with all treatments and tasks. The entire overview of the general experimental/control trials can be found in figure 2.

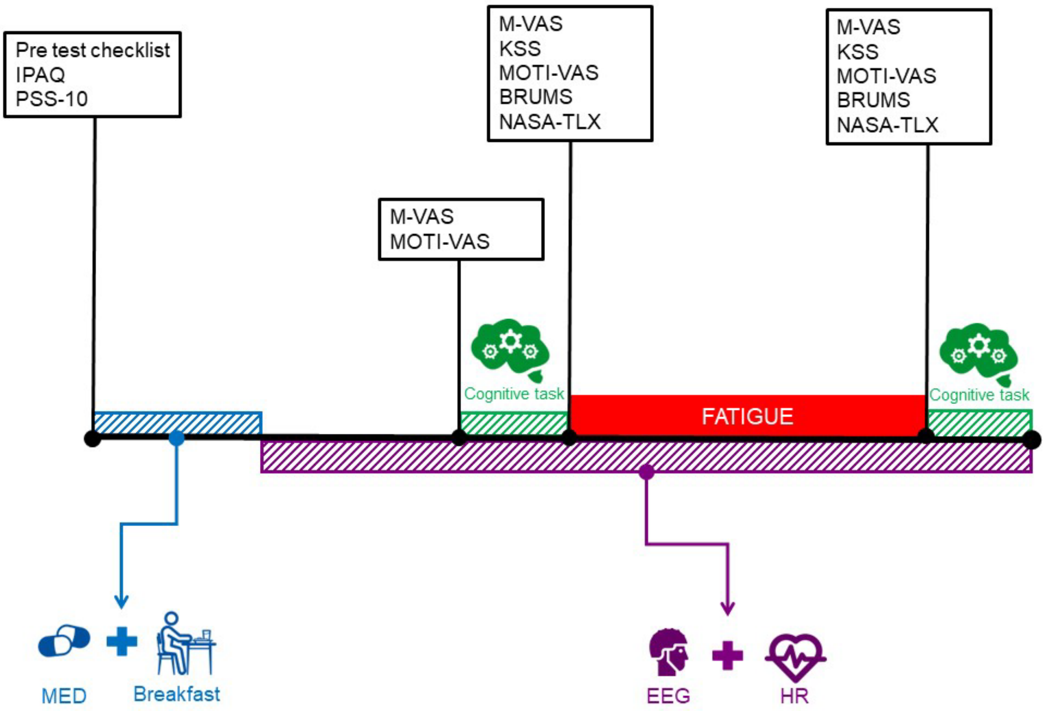

Figure 2: overview of the experimental/control trial

<u>3.3.1. Differences between experimental studies: Induction of Physical, Mental and Combined fatigue</u>

3.3.1.2. Mental Fatigue (experimental study 1)

An overview of the design of this study is shown in figure 3. A modified Stroop task (38, 39), based on their performance during the Stroop max test of approximately 60 min, partitioned in 4 blocks of 360 stimuli (=90 times the different words), will be used as the mentally fatiguing task (see also Habay et al. (2021) (40)). Each word will be presented on screen in 34 point font for 1000 ms with an inter-stimulus interval based on their performance on the max test. Subjects will be instructed to respond as quickly and accurately as possible. To assess performance both ACC and reaction time (RT) will be collected and averaged every block. Each of the 4 blocks will be separated by a 30-s period (where about 15 stimuli were not taken into account), so after every 15 min, during which stimuli will keep presenting themselves, but they will not be counted in the eventual performance outcomes of the different blocks. During this time, participants will be asked verbally about their level of MF from 0 to 100. During each block, EEG and HR data will be assessed and averaged.

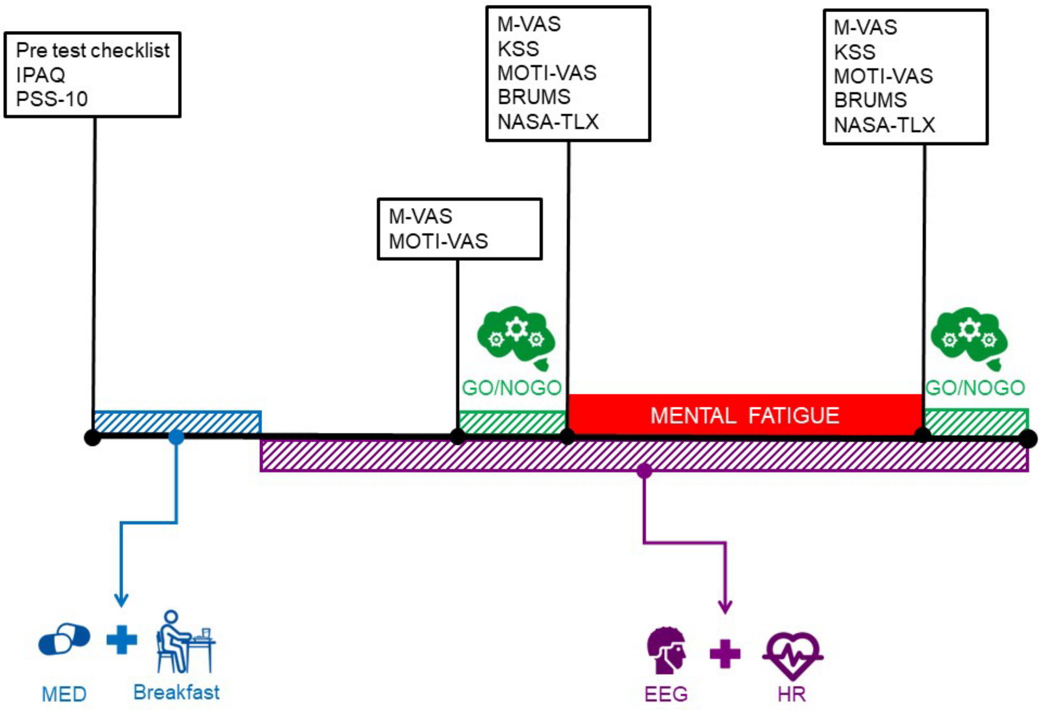

Figure 3. Design of the physical fatigue study

3.3.1.1. Physical Fatigue (experimental study 2)

An overview of the design of this study is shown in figure 4. A leg extension machine will be used to induce PF. The fatiguing protocol is based on the study of Klass et al. (2016) (41) and Proost et al. (2022) (ongoing). Participants first performed a standardized warm up on the machine using a weight of 25 kg. Afterwards, the machine was fitted with the maximal amount of weight that it could hold (i.e. 60 kg), and participants were instructed to perform different leg extensions until failure. The contractions were timed using a visual stimulus provided using the Fitlight (http://www.fitlighttraining.com/) system (a first light to start the contraction, and a second one to finish it). Contractions were dynamic and lasted 2,5 seconds, with 5,5 seconds in between. After performing the contractions until failure, the formula of Lombardi was used to calculate the 1RM measure of the specific participant.

During the intervention session, the PF induction consisted of different contractions at a specific percentage of 1RM until failure. The percentage will probably lie between 40 and 60% (based on pilot testing before the trial). After every 35 contractions (i.e.1 block), a break of 20 seconds was provided where participants reported oral RPE (0-100 scale) and subjective PF (0-100 scale) values. During each block, EEG and HR data will be assessed and averaged. Before and after the physical fatigue task, subjects will perform a psychomotor vigilance and a 15-minute non-invasive tensiomyography (TMG) test of the muscle contractile properties of their lower extremities. Blood lactate and glucose will also be taken before and after the leg extension trial. Task failure was defined as the time when the subject failed to attain the specific contraction for the whole range of motion on three consecutive contractions, with the subjects being informed of each failure (42). Throughout the protocol, no feedback was provided regarding the time lapsed or any physiological parameters.

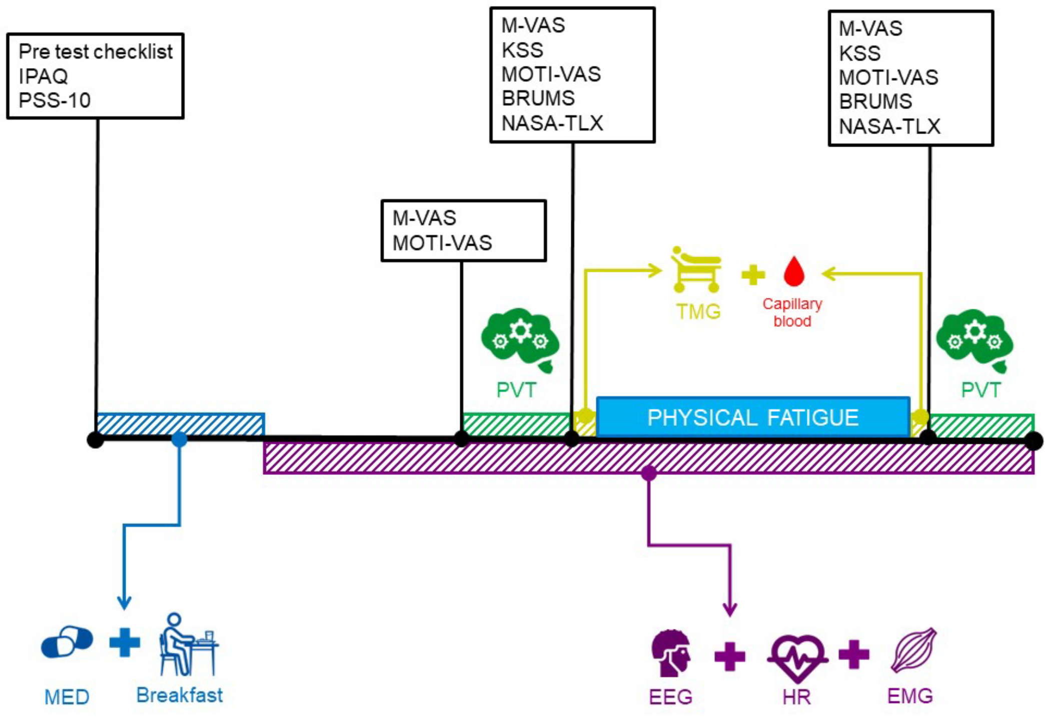

Figure 4: Design of the physical fatigue study

3.3.1.3. Combined fatigue (experimental study 3)

The aim of the combined fatigue trial is to see how neurotransmitters influence the established effects of MF on physical performance (12, 13). The physical and mentally fatiguing protocols (defined in 3.3.1.1. and 3.3.1.2.) will be performed during the same measurement time. Participants will first complete the mentally fatiguing protocol, and will then be instructed to finish the physically fatiguing protocol. The overall design of the trial will therefore also be slightly altered (Figure 5).

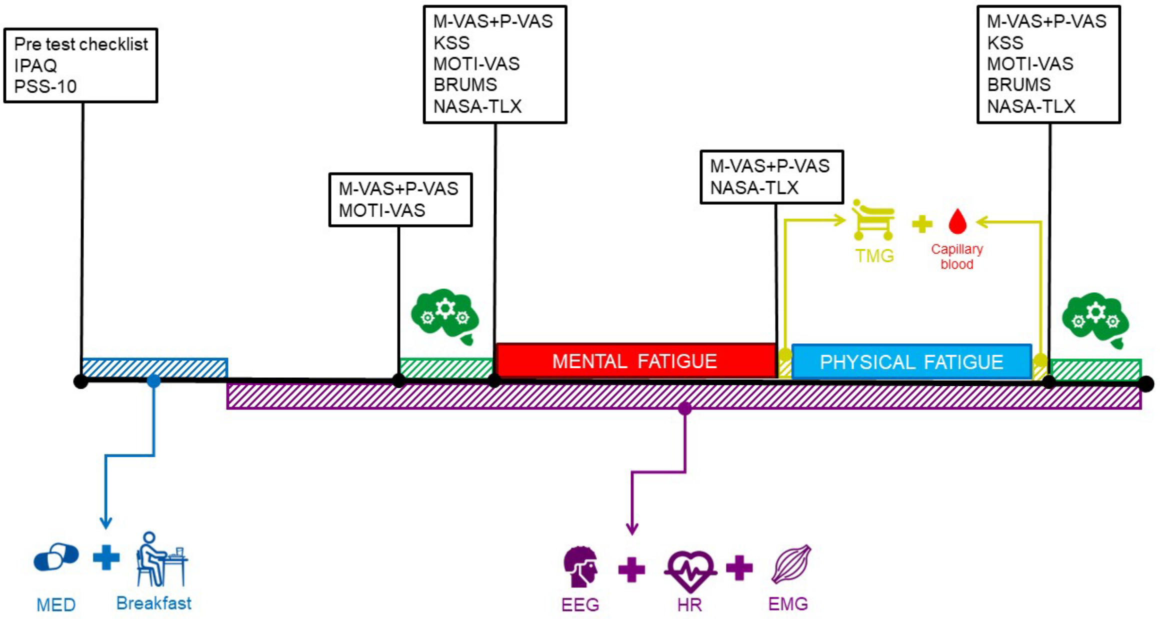

Figure 5: design of the combined fatigue trial

3.3.2. Pharmacological substances:

The utilized medications, their neurotransmitter target and their dosage (along with the reference to substantiate this dosage) are defined under this paragraph. All medications will be administered orally. The important complications for every medication are also defined.

- **Reboxetine (Rebox):** noradrenaline reuptake inhibitor: 8 mg (43).
    - Medicine: Edronax 4 mg
- **Methylphenidate (Mph):** dopamine and noradrenalin reuptake inhibitor: 20 mg (2).
    - Medicine: Rilatine 10 mg

3.3.3. Placebo:

Placebo will be administered using (P-Tabletten weiss 7 mm Lichtenstein): 10 mg.

3.4 Primary outcome measures

3.4.1 Performance measures

The main outcome of each trial will encompass the outcomes that best describe how participants performed during and after the fatiguing protocols. For the PF trials, this outcome is defined as the amount of repetitions until failure. For the MF trial, the level of MF induced will be determined using the outcomes of the Stroop task (reaction time and accuracy) for each block and the M-VAS value

throughout the Stroop task. For the combined fatigue trial, the primary outcome will entail the difference in repetitions until failure due to MF between the different supplements.

Moreover, cognitive performance will also be assessed during each experimental trial and used as a primary outcome measure to assess overall fatigue induced. Cognitive performance will be assessed using a cognitive test battery comprising of the Go/No-Go task (primarily suggested by Guo et al. (44) and Kato et al. (45)) and the PVT task.

- Go/NoGo: Participants are to react to different stimuli displayed on a computer screen positioned about 1 meter from the subject, preceded by a screen with general instructions. Two different stimuli were present at the same time: a Go or NoGo stimuli, and a left or right stimuli (meaning that four combinations are possible: GoRight, GoLeft, NoGoRight and NoGoLeft). If a Go stimuli is presented, participants are instructed to react to the left or right stimuli with the corresponding arrows. However, if a NoGo stimulus is presented, participants are instructed to refrain from reacting to the either the left or right stimuli. This proposed paradigm measures attention, response inhibition and working memory, executive functions who are all consistently negatively impacted by MF (10, 44, 46, 47). Stimuli will be presented for 400 ms, with a varying interstimulus time between 600 and 1400 ms. Outcomes for Go trials include RT and ACC, while the sole outcome for NoGo trials is ACC. The ratio of Go/NoGo trials will be 80 to 20 respectively. Since the trials feature seven different interstimulus times, 1 cycle of trials consists of 112 Go and 28 NoGo trials. Moreover, to consistently detect large ERP's, research has suggested that between 30 and 40 stimuli should be present (48). Therefore, this brings us to a total task duration of about 8,5 min (taken into account a mean trial duration of 1400 ms).
- PVT: The psychomotor vigilance test will be used as a sustained attention task. Briefly, a laptop computer will display a visual stimulus (fireball, always in the middle of the screen) at a variable interval (between 2 and 10 s). Subjects are required to respond by pressing the space bar with the index of the dominant hand. The distance between the subject's eyes and the screen is approximately 40 cm. Subject's reaction time is displayed on a computer screen for 1 s after space bar pressing. If the subject does not respond within 500ms, the trial is stored as a lapse, and a warning message appears to the participant. The PVT runs for a total period of 10 min (100 stimuli), reaction time and number of lapses are recorded.

3.4.2 EEG and EMG

The EEG amplifier device (LiveAmp, Brain Products Munich, Germany) will be used to continuously measure brain activity by using 64 (2x32 channels) paste-free (ThinkPulse) active electrodes attached to the LiveAmp actiCAP adapter.These electrodes will be attached on the participants' head (Acticap slim/snap, Brain Products Munich, Germany), according to the "10:20 International System" (49). The

sampling rate will be set at 500 Hz (Brain Vision Recorder, Brain Products, Munich, Germany). Electrode impedance will be kept <5 kΩ throughout the recording. Baseline measurements will be taken with 10 blinks, 2 min with eyes open and 2 min with eyes closed. During baseline EEG recordings, participants will be seated, have to insert earplugs and will be instructed to minimize movement of the head and eye blinking, to avoid frowning, to maintain the same posture and not to touch their head with their hands in order to minimize movement, sound and muscle artefacts. An automated pre-processing pipeline (filtering, re-referencing, denoising) will precede machine learning processes. EEGlab will be applied to further process the data, i.e. data segmentation (4s segments with at least 10% overlap) and Fast Fourier transform spectral analysis for extracting power spectral data on one hand and continuous data extraction for insertion into the source localization technique standardized low-resolution brain electromagnetic tomography on the other hand. The latter analysis allows us to localize electrocortical alterations (of amplitude, power and current source densities) within the brain.

Electromyography activity will be recorded with the Cometa Wave Plus wireless EMG system). Targeted muscles are the Vastus Lateralis (BL) and Biceps Femoris (BF) muscles, by the use of bipolar silver chloride surface electrodes (20 mm interelectrode distance), during MVC and percutaneous electrical stimulation. Skin impedance will be reduced bellow 5 kOhms by standard preparation including shaving, gentle abrasion and cleaning with an alcohol-based tissue pad. Recording electrodes will be placed along the sagittal axis over the muscle belly, and the reference electrodes onto the medial and lateral tibial condyles. Raw EMG signal will be stored and integrated online with commercially available software (Acqknowledge, Biopac System Inc.). Advanced processing techniques, such as Cumulative Activity Index (CAI) (50) and direct assessment of neural codes in dynamic contractions (51) will be employed to quantify differences in muscle excitations and synergies caused by immobilization in each subject. Quality of extracted information will be strictly assessed (52), enabling accurate insight into adaptations of central control strategies.

3.5. Secondary outcome Measures

3.5.1. Questionnaires during the trial

All questionnaires will be assessed using the research electronic data capture (REDCAP) application(53).

Perceived stress scale. The Perceived Stress Scale (PSS-10) is a 10-item questionnaire originally designed by Cohen (1983)(54) widely used to assess stress levels in people aged 12 and above. The questionnaire evaluates the degree to which an individual has perceived life unpredictable, uncontrollable an overloading over the previous month.

Physical activity. the international physical activity questionnaire short form (IPAQ-SF) will assess the

overall physical activity habits of the participant. The IPAQ-SF consists of 7 questions, assessing amount and level of intensity of physical activity in the last 7 days, and represents good reliability and validity (55, 56)The questionnaire takes approximately 2-3 min to complete.

Mood. The Brunel Mood Scale (BRUMS) will be used to indicate mood, and serve as a control measure for both arousal as well as boredom . It will be assessed before and after each trial. This scale is based on the Profile of Mood States, and was validated using a multi-sample confirmatory factor analysis (57-59). It features 24 mood descriptors (divided into 6 subscales (i.e. anger, confusion, depression, fatigue, tension and vigor)), where participants have to give a value for every mood on a 5-point Likert scale (i.e. 0=not at all to 4 = extremely) responding to the question: “how do you feel right now?” (58). It supports an adequate internal consistency and test-retest reliability (58). Moreover, it has been used in multiple MF and PF studies and can accurately be used as a possible confounder variable (60-62).

Motivation. Motivation will be assessed using a VAS (63). Participants will be asked “How motivated do you feel for the upcoming task”, and will be instructed to rate their answer on a 10cm line, with the statements “not at all” and “extremely motivated” at opposite ends of this line. Since motivation has often been cited as a potential mechanism behind MF (64-66), its measurement might give us a clue about the mechanisms behind possible influencing individual factors. Moreover, multiple MF studies have already used a VAS as a measuring method for motivation

Subjective workload. Subjective workload will be measured with The National Aeronautics and Space Administration Task Load Index (NASA-TLX). The NASA-TLX scale is composed of six subscales assessing subjective workload and will be taken after finishing every performed task during the trial. Amongst other things, the NASA-TLX scale will measure the ability to identify errors. Details about this questionnaire are described by Hart and Staveland, 1988 (67).

Subjective feeling of Mental Fatigue. The M-VAS-scale (0-10cm) will be taken at multiple different time points (68). It will assess how mentally fatigued the subject is feeling (100 point VAS scale), and ranges from ‘not at all’ to ‘completely exhausted’. The validity and reliability of a visual analogue scale to assess general fatigue was demonstrated by Lee et al. (69), and this type of scale has been shown to be one of the most sensitive assessments of MF (70).

Subjective feeling of physical fatigue: a P-VAS scale (0-10 cm) will be administered before and at the end of each time trial to check the level of subjective PF of the participants. Participants will be asked to rate their general PF on a 10 cm line ranging from “not at all” to “completely exhausted”. The validity and reliability of a visual analogue scale to assess general fatigue was demonstrated by Lee et al. (69).

Sleepiness. The Karolinska Sleepiness Scale (KSS) will be conducted before and after the trial. This 10-point scale measures the subjective level of sleepiness and goes from 1 = ‘extremely alert’ to 10 =

'extremely sleepy, falls asleep all the time'. It is an often used scale in sleepiness research (71), Kaida et al. (72) stated that this scale has a high validity in measuring sleepiness.

Subjective feeling of boredom. To assess the feeling of boredom the question "How boring did you find the performed task?" will be asked, the subjects will have to answer on a visual analogue scale that ranges from 'not at all' to 'completely bored'. This visual analogue scale for boredom (B-VAS) will be taken after each cognitive task, the intervention/control trial and the physical task. The validity and reliability of a general visual analogue scale has already been determined (69), and the B-VAS has also already been used in MF research (73).

Internal load: the internal load of the physical performance measure will be examined using the CR100 RPE scale. This scale is a valid and modified version of the original Borg RPE measure, depicting a scale of 1-100 with verbal anchors reading "nothing at all", "minimum", "extremely weak", "very weak", "weak", "moderate", "strong", "very strong", "extremely strong" and "maximal" (74, 75). These verbal anchors are subsequently divided into larger categories (i.e. "just noticeable", "light", "heavy", "max") (74). Moreover, an "absolute maximum" is also depicted, which is displayed far above the so called maximum. The CR100 scale is a reliable and valid measure of internal load in physical performance, and is hypothesized to be more sensitive to changes in internal load compared to the CR10 scale (74, 76).The RPE will be examined before, after and throughout (2 min intervals) the time trial.

3.5.2. Physiological Measurements during the trial

Glucose concentration (mmol/l) (Bayer Contour Next USB Glucosemeter, Leverkusen, Germany) and **Hb** (HemoCue® Hemoglobin Analyzer Meter, Ängelholm, Sweden) will be measured before and after the physical fatiguing tasks. Subject's will be asked to give a 0.6-µl sample of whole blood from the left index finger top for assessment.

Heart Rate will be continuously assessed by using the POLAR H10 sensor (Mental Fatigue). For mental fatigue, heart rate and respiratory rate will be assessed by using Polar H10 and for physical fatigue, the Equivital™ Life Monitor EQ-02 Physiological Status Monitor (PSM) will be used.

Lactate measures: blood lactate will be measured at the beginning and end of the trial at the finger tip of the participant (determined enzymatically; EKF; BIOSEN 5030, Magdeburg, Germany). These measures will only be taken in the physical fatigue studies.

4. Sample size, recruitment and retention interventions

4.1. Mental Fatigue

A priori power analysis ($\eta_p^2 = 0.313$; $\alpha = 0.05$; $\beta = 0.95$ + 10% drop out) indicates we should include 18 healthy, young (18-35), trained subjects. The effect size was calculated from Van Cutsem et al. (2022) (77).

4.2. Physical Fatigue

A priori power analysis (Cohen's d = 0.585; α=0.05; β = 0.95 + 10% drop out) indicates we should include 20 healthy, young (18-35), trained subjects. The effect size was calculated from Roelands et al. (2008) (3).

4.3. Combined Fatigue

A priori power analysis (Hedges g = 0.51; α=0.05; β = 0.95 + 10% drop out) indicates we should include 24 healthy, young (18-35), trained subjects. The effect size was calculated from Brown et al. (2020) (13).

Data collected from participants that dropped out will be deleted and not taken into account in further analysis. Dropout will be reduced by minimizing the time between the different sessions and using additional reminders.

5. Assignment of treatment order and allocation concealment

All participants are required to perform all intervention procedures, and the placebo condition. Treatment order (NA, DA, and PLAC) will be randomized and balanced by using a web-based computer program (www.randomization.com) to determine treatment order.

We will inform the participants in a way that explains the study as an investigation about the involvement of different brain neurotransmitter systems in the onset of physical and mental fatigue. We will strive to induce equal expectations for all the participants in all trials concerning the influence of the different medications on the performance tasks. At the end of the last trial we will ask a standardized question to follow up on their thoughts about the objective of the study. Allocation will not be concealed to trial personnel (outcome assessors and intervention/control providers), since they are in charge of providing participants with the right cognitive task in the middle of the trial. The allocation of participants to specific treatment orders and the generation of these orders will all be performed by the pharmacy of UZ Brussels who will provide us the medications. The allocation list will be shared with two other researchers of our research group who are not involved in this study in any manner. In this way, a triple-blinded study is created in which the researchers, research assistants and participants will be blinded. Treatment allocation will only be reported to the doctor in case of side-effects of the

medication or on specific request (which is always allowed, regardless of the nature of the request). These participants will be excluded from further analysis.

6. Statistical analysis

The person responsible for carrying out the statistical analyses will be Laurisa Arenales Arauz / Tjaša Ocvirk (Human Physiology Research Group, Vrije Universiteit Brussel, Brussels, Belgium). All statistical tests will be conducted in R (version 4.1.2.). All data will be presented as means ± standard deviation (SD). Normality will be checked using the Shapiro-Wilk test, and visually confirmed with histograms. If data is not normally distributed, log and squared data transformations will be performed. If data is still not normally distributed, non-parametrical equivalents will be used instead. The Mauchly's test will be used to verify for sphericity. However, if sphericity cannot be assumed, the Greenhouse-Geisser procedure will be used to correct the significance of F-values. Effect of MF on both physical and cognitive performance will be performed by a 2 (time; only with cognitive performance) x 2 (condition) repeated measures ANOVA. The same base statistical procedure will be used to examine changes in manipulation checks and other psychological and subjective measures during the trial.

7. Data storage and management

7.1. Data storage:

The persons responsible for data management and preservation are Prof. Dr. Bart Roelands. The project will exclusively generate new data.

During the trial, data will be saved on the VUB share point, which has a build in back up. An external back up will be available in the form of an external HDD provided by the MFYS department. After the trial, data will be anonymized where possible and stored in the VUB university archive. An exclusive identity code will be assigned to each participant. The identity of the individual for the exclusive code will only be known by the research team. As the data consists of special categories it will not be stored on any external archives (=restricted access). Option to access the database will require a data use agreement. The stored data will remain unchanged and be identified by the researcher ORCID and article DOI. Transfer of data will only be done if essential. All manual files and written data will be stored in locked filing cabinets.

All anonymized data will be stored for up to four years to allow for access before publication and may be required at a later date for further analysis and auditing. All personal data, including health questionnaires and signed consent forms, will be destroyed within 12 months of the study finishing.

7.2. Data management:

The person responsible for all data management of the present study will be Prof. Dr. Bart Roelands (bart.roelands@vub.be).

The data protection officer of the VUB (dpo@vub.be) will be notified of our study

Data in the present study will be managed confidentially, conform the Algemene Verordening Gegevensbescherming (GDPR) of 27 April 2016. Participating in this study means that the participant agrees that the investigators gather data of the participant, data that will be used to conduct the investigation and eventually will result in the publications in scientific journals and presentations at conferences. The participant will always have the right to ask the investigators which data he/she has gathered on them and for what this data is used in light of the investigation. The participant retains the right to look into this data and to ask for corrections in the occasion this data would contain errors. The participant also retains the right to stop his/her participation in the study at any moment.

In order to control the quality of the present study, it is possible the medical file of the participants is consulted. If so, this will be done by qualified personnel who are bound to professional secrecy, e.g. representatives of the ethical committee or an extern auditing bureau. This can only take place under strict conditions, under the responsibility of the investigator and under his supervision. The encrypted data can also be passed through the Belgium government or another regulatory authority or the ethical committee.

8. Ethics and dissemination

The study will be submitted to the commission of medical ethics at the University Hospital of the Vrije Universiteit Brussel. Moreover, it will also be registered in the Clinicaltrials.gov database.

All alterations of the present protocol, if accepted, will be communicated to the commission of medical ethics through the use of amendments. All of these amendments will be submitted through the Dycoflow system, and subjected to approval by the commission. The proposed changes will only be implemented if they are approved by the mentioned commission.

The approval of subjects to participate in the present trial will be collected using an informed consent which will be designed using a template of the UZBrussel and utilised informed consents from accepted studies at the MFYS research lab. Drs. Laurisa Arenales Arauz / Tjaša Ocvirk will be responsible for providing participants with all required information of the present trial, and making sure that they correctly fill in the afore mentioned forms.

As personal information is branded by the EU as sensitive, data will be anonymized as soon as possible. Participants will receive a code corresponding to their inclusion order, and the file that relates these codes to their personal information will be encrypted. During the course of the entire PhD of Drs. Laurisa Arenales Arauz / Tjaša Ocvirk the data will only be stored with personal access, on a personal cloud

server and an external HDD. After the completion of this PhD, almost all data will be shared with the research lab through a network attached storage, except for the personal data file, which will remain stored on the external HDD. For further details on the preservation of personal data, one can consult the detailed data management plan.

The submitters of the present protocol proposal declare no financial or other conflicts of interest.

Trial results will be communicated to the general public through the publication of disseminated manuscripts published in scientific journals, and through conference presentations/posters. Authorship of these papers and presentations will only be granted to researchers that performed a substantial contribution to the scientific article. Public access will be granted to the entire protocol through the Clinicaltrials.gov registry. The statistical code and dataset will only be shared upon reasonable request, and the responsible data managers reserve the right to refuse such requests.

References

1. Gandevia SC. Spinal and Supraspinal Factors in Human Muscle Fatigue. Physiological Reviews. 2001;81(4):1725-89.
2. Roelands B, Hasegawa H, Watson P, Piacentini MF, Buyse L, De Schutter G, et al. The effects of acute dopamine reuptake inhibition on performance. Med Sci Sports Exerc. 2008;40(5):879-85.
3. Roelands B, Goekint M, Heyman E, Piacentini MF, Watson P, Hasegawa H, et al. Acute norepinephrine reuptake inhibition decreases performance in normal and high ambient temperature. J Appl Physiol (1985). 2008;105(1):206-12.
4. Roelands B, Meeusen R. Alterations in central fatigue by pharmacological manipulations of neurotransmitters in normal and high ambient temperature. 2010. p. 229-46.
5. Habay J, Van Cutsem J, Verschueren J, De Bock S, Proost M, De Wachter J, et al. Mental fatigue and sport-specific psychomotor performance: a systematic review. Sports medicine (accepted for publication). 2021.
6. Boksem MAS, Tops M. Mental fatigue: Costs and benefits. Brain Research Reviews. 2008;59(1):125-39.
7. Marcora SM, Staiano W, Manning V. Mental fatigue impairs physical performance in humans. Journal of applied physiology (Bethesda, Md : 1985). 2009;106(3):857-64.
8. Tran Y, Craig A, Craig R, Chai R, Nguyen H. The influence of mental fatigue on brain activity: Evidence from a systematic review with meta-analyses. Psychophysiology. 2020;57(5).
9. Russell S, Jenkins D, Smith M, Halson S, Kelly V. The application of mental fatigue research to elite team sport performance: New perspectives. Journal of Science and Medicine in Sport. 2019;22(6):723-8.
10. Fan X, Zhou Q, Xie F, Liu Z. Mental Fatigue and Impaired Attention: An Event-Related Potential Study. Journal of Medical Imaging and Health Informatics. 2017;7(2):378-87.
11. van der Linden D, Frese M, Meijman TF. Mental fatigue and the control of cognitive processes: effects on perseveration and planning. Acta psychologica. 2003;113(1):45-65.

12. Van Cutsem J, Marcora S, De Pauw K, Bailey S, Meeusen R, Roelands B. The effects of mental fatigue on physical performance: A systematic review. Sports Medicine. 2017;47(8):1569-88.
13. Brown DMYY, Graham JD, Innes KI, Harris S, Flemington A, Bray SR. Effects of prior cognitive exertion on physical performance: a systematic review and meta-analysis. Sports Medicine. 2020;50(3):497-529.
14. Habay J, Van Cutsem J, Verschueren J, De Bock S, Proost M, De Wachter J, et al. Mental Fatigue and Sport-Specific Psychomotor Performance: A Systematic Review. Sports Medicine. 2021;51(7):1527-48.
15. Johansson B, Wentzel AP, Andrell P, Odenstedt J, Mannheimer C, Ronnback L. Evaluation of dosage, safety and effects of methylphenidate on post-traumatic brain injury symptoms with a focus on mental fatigue and pain. Brain Inj. 2014;28(3):304-10.
16. Moeller SJ, Tomasi D, Honorio J, Volkow ND, Goldstein RZ. Dopaminergic involvement during mental fatigue in health and cocaine addiction. Transl Psychiatry. 2012;2(10):e176.
17. Lorist MM, Bezdan E, ten Caat M, Span MM, Roerdink JB, Maurits NM. The influence of mental fatigue and motivation on neural network dynamics; an EEG coherence study. Brain Res. 2009;1270:95-106.
18. Kok A. Cognitive control, motivation and fatigue: A cognitive neuroscience perspective. Brain Cogn. 2022;160:105880.
19. Lorist MM, Bezdan E, ten Caat M, Span MM, Roerdink JBTM, Maurits NM. The influence of mental fatigue and motivation on neural network dynamics; an EEG coherence study. Brain research. 2009;1270:95-106.
20. Martin K, Meeusen R, Thompson KG, Keegan R, Rattray B. Mental Fatigue Impairs Endurance Performance: A Physiological Explanation. Sports Med. 2018;48(9):2041-51.
21. Van Cutsem J, De Pauw K, Marcora SM, Meeusen R, Roelands B. A caffeine-maltodextrin mouth rinse counters mental fatigue. Psychopharmacology. 2018;235(4):947-58.
22. Jacquet T, Lepers R, Poulin-Charronnat B, Bard P, Pfister P, Pageaux B. Mental fatigue induced by prolonged motor imagery increases perception of effort and the activity of motor areas. Neuropsychologia. 2021;150:107701.
23. Faber LG, Maurits NM, Lorist MM. Mental fatigue affects visual selective attention. PloS one. 2012;7(10):e48073-e.
24. Möckel T, Beste C, Wascher E. The Effects of Time on Task in Response Selection - An ERP Study of Mental Fatigue. Scientific Reports. 2015;5(1):10113.
25. Schmidt EA, Schrauf M, Simon M, Fritzsche M, Buchner A, Kincses WE. Drivers' misjudgement of vigilance state during prolonged monotonous daytime driving. Accid Anal Prev. 2009;41(5):1087-93.
26. Morree HMd, Klein C, Marcora SM. Cortical substrates of the effects of caffeine and time-on-task on perception of effort. Journal of Applied Physiology. 2014;117(12):1514-23.
27. Brownsberger J, Edwards A, Crowther R, Cottrell D. Impact of mental fatigue on self-paced exercise. Int J Sports Med. 2013;34(12):1029-36.
28. Blankertz B, Tomioka R, Lemm S, Kawanabe M, Muller Kr. Optimizing Spatial filters for Robust EEG Single-Trial Analysis. IEEE Signal Processing Magazine. 2008;25(1):41-56.
29. Ehinger BV, Dimigen O. Unfold: an integrated toolbox for overlap correction, non-linear modeling, and regression-based EEG analysis. PeerJ. 2019;7:e7838.

30. Dahne S, Meinecke FC, Haufe S, Hohne J, Tangermann M, Muller KR, et al. SPoC: a novel framework for relating the amplitude of neuronal oscillations to behaviorally relevant parameters. Neuroimage. 2014;86:111-22.
31. Shine JM, Breakspear M, Bell PT, Ehgoetz Martens KA, Shine R, Koyejo O, et al. Human cognition involves the dynamic integration of neural activity and neuromodulatory systems. Nature Neuroscience. 2019;22(2):289-96.
32. Chan A-W, Tetzlaff JM, Gotzsche PC, Altman DG, Mann H, Berlin JA, et al. SPIRIT 2013 explanation and elaboration: guidance for protocols of clinical trials. BMJ. 2013;346(jan08 15):e7586-e.
33. Association AP. American Psychiatric Association: Diagnostic and Statistical Manual of Mental Disorders, 5th Edition. Arlington, VA2013.
34. Schaufeli WB, Desart S, De Witte H. Burnout Assessment Tool (BAT)—Development, Validity, and Reliability. International Journal of Environmental Research and Public Health. 2020;17(24):9495.
35. Smets EMA, Garssen B, Bonke B, De Haes JCJM. The multidimensional fatigue inventory (MFI): psychometric qualities of an instrument to assess fatigue. Journal of Psychosomatic Research. 1995;39(5):315-25.
36. Wang Y-P, Gorenstein C. Psychometric properties of the Beck Depression Inventory-II: a comprehensive review. Revista Brasileira de Psiquiatria. 2013;35(4):416-31.
37. Arnau S, Brummer T, Liegel N, Wascher E. Inverse effects of time-on-task in task-related and task-unrelated theta activity. Psychophysiology. 2021;58(6):e13805.
38. Smith MR, Coutts AJ, Merlini M, Deprez D, Lenoir M, Marcora SM. Mental Fatigue Impairs Soccer-Specific Physical and Technical Performance. Medicine and science in sports and exercise. 2016;48(2):267-76.
39. Pageaux B, Marcora SM, Rozand V, Lepers R. Mental fatigue induced by prolonged self-regulation does not exacerbate central fatigue during subsequent whole-body endurance exercise. Front Hum Neurosci. 2015;9:67.
40. Habay J, Proost M, De Wachter J, Díaz-García J, De Pauw K, Meeusen R, et al. Mental Fatigue-Associated Decrease in Table Tennis Performance: Is There an Electrophysiological Signature? International Journal of Environmental Research and Public Health. 2021;18(24):12906.
41. Klass M, Duchateau J, Rabec S, Meeusen R, Roelands B. Noradrenaline Reuptake Inhibition Impairs Cortical Output and Limits Endurance Time. Med Sci Sports Exerc. 2016;48(6):1014-23.
42. Burnley M, Vanhatalo A, Jones AM. Distinct profiles of neuromuscular fatigue during muscle contractions below and above the critical torque in humans. J Appl Physiol (1985). 2012;113(2):215-23.
43. Klass M, Roelands B, Meeusen R, Duchateau J. Acute Effect of Noradrenergic Modulation on Motor Output Adjustment in Men. Med Sci Sports Exerc. 2018;50(8):1579-87.
44. Guo Z, Chen R, Liu X, Zhao G, Zheng Y, Gong M, et al. The impairing effects of mental fatigue on response inhibition: An ERP study. PloS one. 2018;13(6):e0198206-e.
45. Kato Y, Endo H, Kizuka T. Mental fatigue and impaired response processes: Event-related brain potentials in a Go/NoGo task. International Journal of Psychophysiology. 2009;72(2):204-11.
46. Hofmann W, Schmeichel BJ, Baddeley AD. Executive functions and self-regulation. Trends Cogn Sci. 2012;16(3):174-80.

47. Fan X, Zhao C, Luo H, Zhang W. The Effect of Mental Fatigue on Response Processes: An ERP Study in Go/NoGo Task. In: Ayaz H, Mazur L, editors. Advances in Neuroergonomics and Cognitive Engineering. Advances in Intelligent Systems and Computing. 7752019. p. 49-60.
48. Luck SJ. An Introduction to the Event-Related Potential Technique, second edition. Cambridge, MA, United States: The MIT press; 2014.
49. Jasper H. Report of the committee on methods of clinical examination in electroencephalography. Electroencephalography and Clinical Neurophysiology,. 1958;10:370-1.
50. Kranjec J, Holobar A. Improved Assessment of Muscle Excitation From Surface Electromyograms in Isometric Muscle Contractions. IEEE Trans Neural Syst Rehabil Eng. 2019;27(7):1483-91.
51. Glaser V, Holobar A. Motor Unit Identification From High-Density Surface Electromyograms in Repeated Dynamic Muscle Contractions. IEEE Trans Neural Syst Rehabil Eng. 2019;27(1):66-75.
52. Urh F, Holobar A. Automatic Identification of Individual Motor Unit Firing Accuracy From High-Density Surface Electromyograms. IEEE Trans Neural Syst Rehabil Eng. 2020;28(2):419-26.
53. Harris PA, Taylor R, Thielke R, Payne J, Gonzalez N, Conde JG. Research electronic data capture (REDCap)--a metadata-driven methodology and workflow process for providing translational research informatics support. J Biomed Inform. 2009;42(2):377-81.
54. Cohen S, Kamarck, T., & Mermelstein, R. A global measure of perceived stress. . Journal of health and social behavior. 1983;4(24):385-96.
55. Craig CL, Marshall AL, Sjostrom M, Bauman AE, Booth ML, Ainsworth BE, et al. International physical activity questionnaire: 12-country reliability and validity. Med Sci Sports Exerc. 2003;35(8):1381-95.
56. Lee PH, Macfarlane, D. J., Lam, T. H., & Stewart, S. M. . Validity of the International Physical Activity Questionnaire Short Form (IPAQ-SF): a systematic review. The international journal of behavioral nutrition and physical activity. 2011;8:115.
57. Terry P, Lane A, Fogarty G. Construct validity of the Profile of Mood States — Adolescents for use with adults. Psychology of Sport and Exercise. 2003;4:125-39.
58. Parsons-Smith RL, Terry PC, Machin MA. Identification and Description of Novel Mood Profile Clusters. Front Psychol. 2017;8:1958.
59. Terry PC, Lane AM, Lane HJ, Keohane L. Development and validation of a mood measure for adolescents. Journal of Sports Sciences. 1999;17(11):861-72.
60. Filipas L, Mottola F, Tagliabue G, La Torre A. The effect of mentally demanding cognitive tasks on rowing performance in young athletes. Psychology of Sport and Exercise. 2018;39:52-62.
61. Holgado D, Troya E, Perales JC, Vadillo MA, Sanabria D. Does mental fatigue impair physical performance? A replication study. European journal of sport science. 2020:1-23.
62. Znazen H, Slimani M, Hadadi A, Alzahrani T, Tod D, Bragazzi NL, et al. Acute Effects of Moderate versus High-Intensity Strength Exercise on Attention and Mood States in Female Physical Education Students. Life. 2021;11(9):931.
63. Wewers ME, Lowe N. A critical review of visual analogue scales in the measurement of clinical phenomena. Research in nursing & health. 1990;13 4:227-36.
64. Herlambang MB, Taatgen NA, Cnossen F. The Role of Motivation as a Factor in Mental Fatigue. Human factors. 2019;61(7):1171-85.

65. Meeusen R, Van Cutsem J, Roelands B. Endurance exercise-induced and mental fatigue and the brain. Experimental physiology. 2020;106:2294-8.

66. Martin K, Meeusen R, Thompson KG, Keegan R, Rattray B. Mental fatigue impairs endurance performance: a physiological explanation. Sports Medicine. 2018;48(9):2041-51.

67. Hart SG, Staveland, L.E. Development of NASA-TLX (task load index): results of empirical and theoretical research. Human Ment Workload. 1988;1:139-83.

68. Smith MR, Chai R, Nguyen HT, Marcora SM, Coutts AJ. Comparing the Effects of Three Cognitive Tasks on Indicators of Mental Fatigue. J Psychol. 2019;153(8):759-83.

69. Lee KA, Hicks G, Nino-Murcia G. Validity and reliability of a scale to assess fatigue. Psychiatry Research. 1991;36(3):291-8.

70. Smith MR, Chai R, Nguyen HT, Marcora SM, Coutts AJ. Comparing the effects of three cognitive tasks on indicators of mental fatigue. The Journal of psychology. 2019;153(8):759-83.

71. Short MA, Agostini A, Lushington K, Dorrian J. A systematic review of the sleep, sleepiness, and performance implications of limited wake shift work schedules. Scand J Work Environ Health. 2015;41(5):425-40.

72. Kaida K, Takahashi M, Akerstedt T, Nakata A, Otsuka Y, Haratani T, et al. Validation of the Karolinska sleepiness scale against performance and EEG variables. Clin Neurophysiol. 2006;117(7):1574-81.

73. Mangin T, André N, Benraiss A, Pageaux B, Audiffren M. No ego-depletion effect without a good control task. Psychology of Sport and Exercise. 2021;57.

74. Borg E, Kaijser L. A comparison between three rating scales for perceived exertion and two different work tests. Scandinavian Journal of Medicine and Science in Sports. 2006;16(1):57-69.

75. Borg GAV. Psychophysical bases of perceived exertion. Medicine and Science in Sports and Exercise. 1982;14(5):377-81.

76. Coyne JOC, Gregory Haff G, Coutts AJ, Newton RU, Nimphius S. The Current State of Subjective Training Load Monitoring-a Practical Perspective and Call to Action. Sports Med Open. 2018;4(1):58.

77. Van Cutsem J, Van Schuerbeek P, Pattyn N, Raeymaekers H, De Mey J, Meeusen R, et al. A drop in cognitive performance, whodunit? Subjective mental fatigue, brain deactivation or increased parasympathetic activity? It's complicated! Cortex. 2022;155:30-45.
