# Supplementary material for: The interplay of brain neurotransmission and mental fatigue: A research protocol
Source: PLoS One. 2024 Sep 10;19(9):e0310271. doi: 10.1371/journal.pone.0310271 (PMC11386444; doi:10.1371/journal.pone.0310271)
Supplement: S3 File — (PDF) [file pone.0310271.s003.pdf]

S3. Randomization of medication order

Participant nummer	Trial 1	Trial 2	Trial 3
**1**	1	3	2
**2**	2	3	1
**3**	2	1	3
**4**	1	2	3
**5**	3	1	2
**6**	3	1	2
**7**	1	2	3
**8**	2	3	1
**9**	2	1	3
**10**	3	2	1
**11**	3	1	2
**12**	2	3	1
**13**	3	2	1
**14**	2	1	3
**15**	1	2	3
**16**	1	3	2
