# Supplementary material for: The interplay of brain neurotransmission and mental fatigue: A research protocol
Source: PLoS One. 2024 Sep 10;19(9):e0310271. doi: 10.1371/journal.pone.0310271 (PMC11386444; doi:10.1371/journal.pone.0310271)
Supplement: S4 File — (PDF) [file pone.0310271.s004.pdf]

S4. Standardized breakfast

Cornflakes

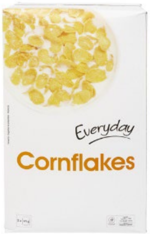

PORTIE	KH (g)	VET (g)	EIWIT (g)	KCAL
100g	82	0.8	8	373

Halfvolle melk

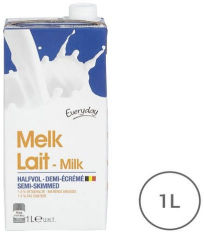

PORTIE	KH (g)	VET (g)	EIWIT (g)	KCAL
150ml	7.5	2.25	5.25	72

Platte kaas met fruit

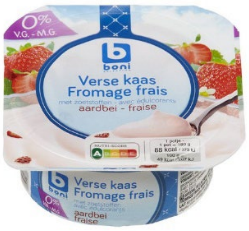

PORTIE	KH (g)	VET (g)	EIWIT (g)	KCAL
180g	9.4	0	11.34	88.2

Sinaasappelsap

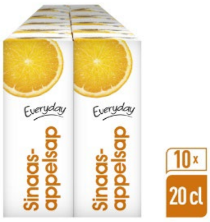

PORTIE	KH (g)	VET (g)	EIWIT (g)	KCAL
200ml	18.2	<1g	1.4	84

Totaal

KH (g)	VET (g)	EIWIT (g)	KCAL
117.1	3.05	25.99	617.2
80%	2%	18%	
